# Hidden among Sea Anemones: The First Comprehensive Phylogenetic Reconstruction of the Order Actiniaria (Cnidaria, Anthozoa, Hexacorallia) Reveals a Novel Group of Hexacorals

**DOI:** 10.1371/journal.pone.0096998

**Published:** 2014-05-07

**Authors:** Estefanía Rodríguez, Marcos S. Barbeitos, Mercer R. Brugler, Louise M. Crowley, Alejandro Grajales, Luciana Gusmão, Verena Häussermann, Abigail Reft, Marymegan Daly

**Affiliations:** 1 Division of Invertebrate Zoology, American Museum of Natural History, New York City, New York, United States of America; 2 Sackler Institute for Comparative Genomics, American Museum of Natural History, New York City, New York, United States of America; 3 Departamento de Zoologia, Universidade Federal do Paraná, Curitiba, Brazil; 4 Richard Gilder Graduate School, American Museum of Natural History, New York City, New York, United States of America; 5 Departamento de Zoologia, Universidade de São Paulo, São Paulo, Brazil; 6 Escuela de Ciencias del Mar, Pontificia Universidad Católica de Valparaíso, Valparaíso, Chile; 7 Department of Molecular Evolution and Genomics, University of Heidelberg, Heidelberg, Germany; 8 Department of Evolution, Ecology, and Organismal Biology, Ohio State University, Columbus, Ohio, United States of America; Australian Museum, Australia

## Abstract

Sea anemones (order Actiniaria) are among the most diverse and successful members of the anthozoan subclass Hexacorallia, occupying benthic marine habitats across all depths and latitudes. Actiniaria comprises approximately 1,200 species of solitary and skeleton-less polyps and lacks any anatomical synapomorphy. Although monophyly is anticipated based on higher-level molecular phylogenies of Cnidaria, to date, monophyly has not been explicitly tested and at least some hypotheses on the diversification of Hexacorallia have suggested that actiniarians are para- or poly-phyletic. Published phylogenies have demonstrated the inadequacy of existing morphological-based classifications within Actiniaria. Superfamilial groups and most families and genera that have been rigorously studied are not monophyletic, indicating conflict with the current hierarchical classification. We test the monophyly of Actiniaria using two nuclear and three mitochondrial genes with multiple analytical methods. These analyses are the first to include representatives of all three currently-recognized suborders within Actiniaria. We do not recover Actiniaria as a monophyletic clade: the deep-sea anemone *Boloceroides daphneae,* previously included within the infraorder Boloceroidaria, is resolved outside of Actiniaria in several of the analyses. We erect a new genus and family for *B. daphneae*, and rank this taxon *incerti ordinis*. Based on our comprehensive phylogeny, we propose a new formal higher-level classification for Actiniaria composed of only two suborders, Anenthemonae and Enthemonae. Suborder Anenthemonae includes actiniarians with a unique arrangement of mesenteries (members of Edwardsiidae and former suborder Endocoelantheae). Suborder Enthemonae includes actiniarians with the typical arrangement of mesenteries for actiniarians (members of former suborders Protantheae, Ptychodacteae, and Nynantheae and subgroups therein). We also erect subgroups within these two newly-erected suborders. Although some relationships among these newly-defined groups are still ambiguous, morphological and molecular results are consistent enough to proceed with a new higher-level classification and to discuss the putative functional and evolutionary significance of several morphological attributes within Actiniaria.

## Introduction

Sea anemones (order Actiniaria) are among the most diverse and successful members of the anthozoan subclass Hexacorallia, occupying benthic marine habitats across all depths and latitudes. Compared to other members of Anthozoa or Hexacorallia, actiniarians show great diversity in polyp anatomy; mesentery arrangement, musculature, column morphology, and tentacle morphology all vary within this group. Furthermore, actiniarians display remarkably diverse life history strategies and engage in symbioses with photosymbiotic microorgansisms and with metazoans including sponges, crustaceans, and fish. The only unique phenotypic features identified so far for members of the order are apical flaps on the nematocysts, three triangular elements in the apex of the capsules that flex outward during discharge [1].

Because anemones are characterized by an absence of attributes that define other hexacorallian orders and are so diverse anatomically, it has been hypothesized that the order is paraphyletic (e.g., [2], [3]). Although monophyly is anticipated based on broad-scale studies of intra-cnidarian relationships (actiniarians are typically resolved as the sister group to hexacorallian orders Antipatharia, Corallimorpharia, Scleractinia and Zoanthidea) [4]–[7], these broad-scale studies emphasized relationships among anthozoans and therefore did not sample sea anemones well enough to provide a compelling test of actiniarian monophyly. Furthermore, studies of relationships among sea anemones have only focused on the suborder Nynantheae, the most species-rich suborder of the three currently recognized within Actiniaria [8]–[11] and thus have not addressed relationships among the suborders within Actiniaria.

The current classification of Actiniaria derives from that of Carlgren [12], which was modified from an earlier classification by Stephenson [13]. This system recognizes three suborders: Endocoelantheae, Nynantheae, and Protantheae [14]. A fourth suborder was introduced by Cappola and Fautin [15], who re-classified as suborder Ptychodacteae what Carlgren [12] considered order Ptychodactiaria. Nynantheae includes most of the known and common species (∼1,100) and is heterogeneous in terms of the anatomy, life history, and ecology of its members. In contrast, Endocoelantheae, Protantheae, and Ptychodacteae are each relatively homogenous [12] and species-poor [14].

Because each subordinal group in Actiniaria is recognized by a unique feature or the absence of a feature characterizing the other three, this classification system implies no clear relationship among the suborders. The four suborders are defined by ciliated tracts in the filaments (present in Endocoelantheae and Nynantheae, absent in Protantheae and Pychodacteae) and arrangement of mesenteries [12]; Endocoelantheae and Ptychodacteae each have unique mesenteries, arranged unlike those of Nynantheae and Protantheae [12], [15].

Carlgren’s [12] division of the suborders also includes muscles at the base of the animal ( = basilar muscles), but as these are absent in members of Endocoelantheae, Protantheae, and Ptychodacteae and in several groups within Nynantheae (described as “with or without muscles”: Carlgren [12]: p. 17), the value of this feature for his subordinal classification is unclear.

Regardless of its value as a feature for subordinal grouping, basilar muscles are of central importance to Carlgren’s [12] division of Nynantheae, being critical in his diagnoses of its three infraorders (erroneously called tribes, see [16]) Athenaria, Boloceroidaria, and Thenaria. Members of Athenaria and Boloceroidaria lack basilar muscles [12]; although these muscles are characterized as “present” in members of Thenaria ( [12]: p. 21), their occurrence varies within this group (e.g., Actinodendronidae, *Andresia,* and *Bathyphellia* lack them, see [17], [13], [18], respectively), essentially rendering Thenaria indistinguishable from the basilar muscle-less Athenaria in practice (see [13]). In contrast, Boloceroidaria is more narrowly defined, and its members have discrete morphological attributes, including a column with longitudinal ectodermal muscles; in addition, the tentacles of members of this infraorder are usually deciduous with endodermal sphincters at the base of each [12], [13].

Members of the infraorder Athenaria are characterized by similarities in gross morphology and habit; this infraorder contains burrowing sea anemones with an elongated column and with a round aboral end that lacks basilar musculature. These attributes have apparently arisen (or been lost) multiple times within Nynantheae [6], [8], [11], [19]. As in many members of Thenaria, in some members of Athenaria the endodermal circular muscles are concentrated distally into a band called the marginal sphincter [20]. This muscle encircles the distal column and allows a polyp to constrict its diameter. It is referred to as “endodermal” when the muscle fibers are on the gastrodermal side of the mesoglea or “mesogleal” when the muscles fibers and cells are embedded in the mesoglea. Carlgren [12] used the marginal sphincter muscle as a primary feature for distinguishing among members of Thenaria; he recognized three unranked groups–Endomyaria, Mesomyaria, and Acontiaria–based largely on whether the sphincter is endodermal (Endomyaria) or mesogleal (Mesomyaria and Acontiaria). Acontiarians differ from mesomyarians in having nematocyst-dense threads called acontia on the mesenteries (reviewed in [11]). As with the other groups he recognized, Carlgren’s Endomyaria, Mesomyaria, and Acontiaria are problematic in practice because they fail to accurately describe the diversity of nynantheans (reviewed in [11]).

Phylogenetic analyses have highlighted the inadequacy of existing morphology-based classifications of Actiniaria. Ptychodacteae has been repeatedly recovered as nested within Nynantheae (e.g., [7], [8], [11]). Furthermore, none of the superfamilial groupings within Nynantheae are monophyletic [8], [11], and the division of the suborder Nynantheae into the infraorders Athenaria, Boloceroidaria, and Thenaria is inadequate [7], [8], [10], [11]. Rather than representing equivalent groups, the three nynanthean infraorders are nested, such that Athenaria and Boloceroidaria lie within Thenaria. Although some rearrangements have been made in the classification of the order based on molecular analyses (e.g., erection of the superfamily Metridioidea to unite acontiate lineages, some lineages that have lost this attribute, and some lineages previously classified within Athenaria–see [11]), these all focus on Nynantheae, leaving monophyly and higher-level relationships within the order untested.

We apply multiple analytical methods to a dataset of five molecular markers (three mitochondrial and two nuclear) for 156 taxa to test the monophyly and higher-level relationships of the order Actiniaria. Based on our results, we propose a new higher-level classification for Actiniaria and consider the putative functional and evolutionary significance of several morphological attributes of groups within Actiniaria.

## Materials and Methods

### Taxonomic Sampling

No special specific permit was required to collect marine anemones. The sampling did not involve endangered or protected species. Actiniarian specimens were collected intertidally by hand, through SCUBA diving, or via trawls. All specimens were identified using polyp anatomy and the distribution and size of cnidae in various regions of the polyp. Voucher specimens fixed in formalin have been deposited at the American Museum of Natural History (AMNH), the Bavarian State Collection of Zoology (ZSM), the California Academy of Sciences (CAS), the Field Museum of Natural History (FMNH), the Rijksmuseum van Natuurlijke Historie (RMNH), the University of Kansas Natural History Museum (KUNHM), and the U. S. National Museum of Natural History (USNM).

We provide new DNA sequence data for multiple representatives of each of the historically-proposed higher-level groups within Actiniaria, representing approximately three quarters (67%) of the family-level diversity within the order (Table S1). Specimens from multiple localities were sampled for broadly distributed or potentially heterogeneous taxa such as *Metridium senile* [21] or *Diadumene* ( = *Haliplanella*) *lineata* [22]. Three species within the subclass Octocorallia–the putative sister group to the subclass Hexacorallia ([23], [24]; but see [25])–were used as outgroups. To test the monophyly of the order Actiniaria rigorously, we included taxa representing the breadth of hexacorallian diversity, including multiple species from the orders Antipatharia, Ceriantharia, Corallimorpharia, Scleractinia, and Zoanthidea. We only included taxa for which we were able to amplify at least three of the five markers used, which resulted in a phylogeny consisting of 156 taxa. DNA sequences from GenBank were also included as appropriate (Table S1). Note that in a few cases, not all markers have been sequenced for the same individual within a species or the same specimens within a genus. Thus, the combined analysis contains terminal taxa that are chimeric at some level (e.g., *Cirrhipathes*, *Aiptasia pallida* [26], *Dendronephthya, Pavona*); these chimeras are indicated with asterisks in Table S1.

We have defined and listed the taxonomic changes made in this work in Appendix 1. Authorship of new names here contained should be attribute to Rodríguez and Daly. We have omitted authorship information for taxa above the level of species within the text; refer to Appendix 1, [14], [27], [28] for this information.

The electronic edition of this article conforms to the requirements of the amended International Code of Zoological Nomenclature (ICZN), and hence the new names contained herein are available under that Code from the electronic edition of this article. This published work and the nomenclatural acts it contains have been registered in ZooBank, the online registration system for the ICZN. The ZooBank LSIDs (Life Science Identifiers) can be resolved and the associated information viewed through any standard web browser by appending the LSID to the prefix “http://zoobank.org/”. The LSID for this publication is: urn:lsid:zoobank.org:pub:F8A2D3D5-AF8D-4870-8902-0D81FB02840B. The electronic edition of this work was published in a journal with an ISSN, and has been archived and is available from the following digital repositories: PubMed Central, LOCKSS.

### Data Collection

Total genomic DNA was isolated from tentacle or column tissue using the Qiagen DNeasy Blood and Tissue Kit, or by standard CTAB extraction [29]. Whole genomic DNA was amplified using published primers while applying standard PCR techniques [30]. We specifically targeted three mitochondrial (partial 12S rDNA, 16S rDNA and *cox*3) and two nuclear (18S rDNA and partial 28S rDNA) markers for phylogenetic reconstruction (see their application in [8], [9], [11]). Samples that could not be readily amplified using standard protocols were amplified with the high-fidelity enzyme Herculase (Stratagene, La Jolla, CA) using manufacturer supplied protocols. All PCR products were cleaned using AmPure magnetic bead solution (AgenCourt, Danvers, MA) and rehydrated with deionized, double-distilled water. Cycle sequencing reactions used.0.6–5.0 µL of purified PCR product, at a concentration of 25 ng of PCR product for every 200 base pairs of length. Cleaned cycle sequence products (using AmPure and/or CleanSeq) were sequenced via traditional Sanger-based capillary electrophoresis on an ABI 3730*xl* by staff at the sequencing facilities of Beckman-Coulter (Danvers, MA) and at the in-house sequencing facilities of the AMNH. Forward and reverse sequences were assembled in Sequencher v.4.9 (Gene Codes Corporation, Ann Arbor, MI) and compared (via BLAST) against the nucleotide database of GenBank to determine whether the target locus and organism were sequenced rather than a symbiont or other contaminant. Given the unexpected position of *Boloceroides daphneae* [31] in our initial phylogenetic reconstructions, we extracted and re-sequenced the DNA of two specimens a total of four times to ensure that the results were not spurious. All sequences have been deposited in GenBank (Table S1).

### Data Analysis

We conduted several analytical methods of phylogenetic inference. We present a summary here for clarity and simplicity; we refer to Text S1 for full details on the all methods.

DNA sequences for each marker were separately aligned using MAFFT v.6.815 [32] using the following settings and parameters: Strategy, L-INS-i (recommended for <200 sequences with one conserved domain and long gaps); Scoring matrix for nucleotide sequences, 200PAM/k = 2; Gap opening penalty, 1.53; Offset value, 0.05; Max iterate, 1000; Retree, 1. Additionally, marker-specific multiple sequence alignments were analyzed with Gblocks [33] to remove poorly-aligned and/or divergent regions. The following parameters were implemented: Maximum number of contiguous non-conserved positions, 8; Minimum length of a block, 5; Gap positions allowed.

Secondary structure (SS) alignments were produced for 12S, 18S and 28S. See Text S1 for details on how the SS alignments were produced.

Complete and reduced (Gblocks) alignments for each marker were analyzed separately and as a concatenated dataset. The reduced concatenated alignment, which was used for all phylogenetic reconstructions (except those analyzed with POY), was deposited in TreeBase (http://www.treebase.org/treebase/index.html).

#### Parsimony

The Incongruence Length Difference test (ILD: [34], [35]) was used to identify instances of incongruence among and between the nuclear and mitochondrial markers. Tree searches under maximum parsimony were conducted using random and consensus sectorial searches, tree drifting, and 100 rounds of tree fusing in TNT v.1.1 [36]. In all these analyses, gaps (-) were treated as missing data. Trees of minimum length were found at least five times. The combined data were subjected to 1000 rounds of bootstrap resampling to assess support for clades.

Heuristic searches were also performed in POY v.4.1.3. [37] using a timed search. See Text S1 for details of these analyses.

#### Model-based

Maximum likelihood (ML): Maximum likelihood analyses were performed in RAxML v.7.6.3 [38] implemented on the CIPRES Science Gateway Portal [39] using the GTR+Γ (-GTRGAMMA) option as model of substitution, but allowing estimation of α shape, GTR rates and base frequencies for each marker in the combined alignment. The *cox*3 alignment was partitioned by codon position. Clade support for the reduced concatenated data set was conducted using rapid bootstrapping (RB) with a subsequent ML search and let RAxML halt bootstrapping automatically (using MRE-based bootstopping criterion).

PartitionFinder [40] was used to select models for each partition in the analysis of the concatenated dataset including SS alignments.

Bayesian: Analyses were performed using MrBayes v.3.1.2 [41]–[43] and PhyloBayes v.3.3b [44]. See Tables S2–4 regarding run parameters and runs.

#### Ancestral state reconstruction

Maximum parsimony ancestral state reconstructions were performed using Mesquite v.1.1 [45] for seven morphological characters (acontia, basilar muscles, deciduous tentacles, endosymbionts, longitudinal ectodermal muscles in the column, marginal sphincter muscle, nematocysts with apical flaps: Table S5) at all internal nodes of the phylogenetic tree derived from the ML analysis. Nodes with <50% bootstrap support were collapsed for the reconstructions.

## Results

### Nodal Support Based on Our Current Molecular Markers

**Figure 1:**
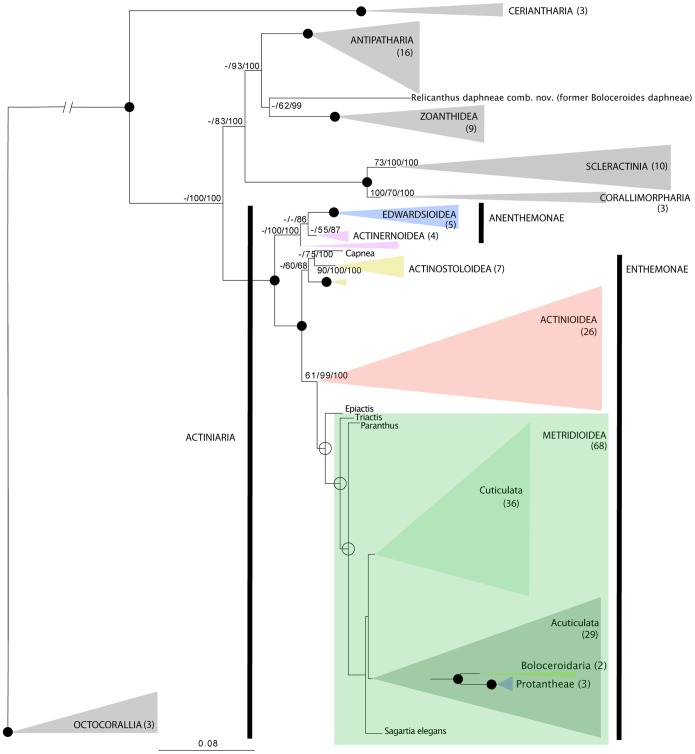
Phylogenetic reconstruction of Hexacorallia. Tree resulting from the Maximum Likelihood (ML) analysis of concatenated 12S, 16S, 18S, 28S and *cox*3. Grey triangles indicate hexacoral orders other than Actiniaria; breadth of triangles corresponds with branch lengths. Species epithets are given only for actiniarian genera represented by more than one species. Numbers above the branches are bootstrap resampling values (parsimony [TNT] and ML, respectively) expressed as a percent, followed by posterior probabilities (multiplied by 100 for legibility); values <50 indicated by “–”; filled-in circles indicate nodes with support of 100% for all inferences; empty circles indicate nodes with support of 100% for posterior probablilities but not support for parsimony and ML. Colored shaded boxes indicate clades defined in the text; the name of each clade is next to or inside the shaded box; the number next to or below the clade name indicates the number of taxa included; branches leading to members of former Protanteae and Boloceroidaria shown.

**Figure 2:**
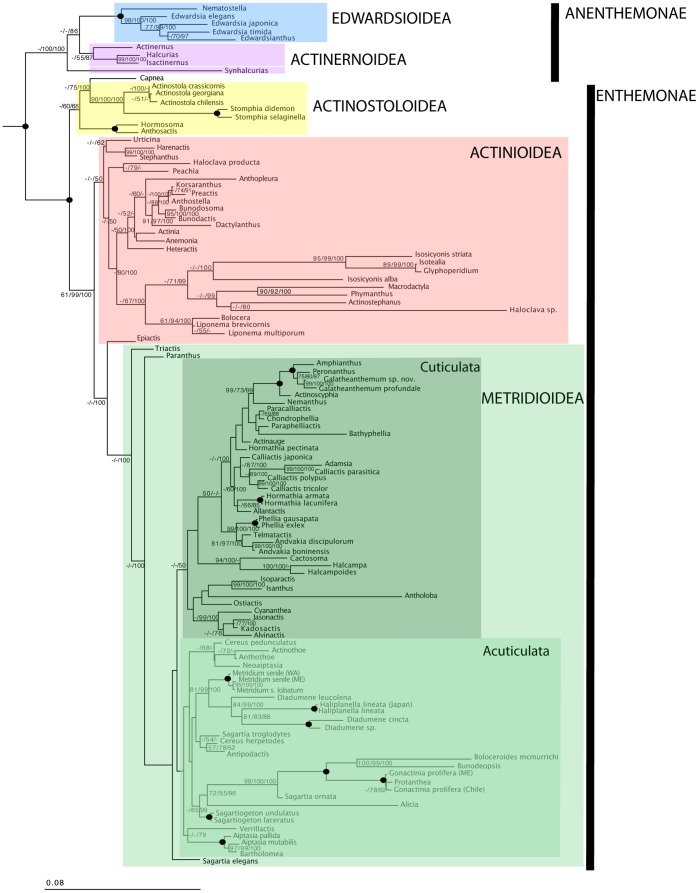
Phylogenetic reconstruction of Actiniaria. Tree resulting from the Maximum Likelihood (ML) analysis of concatenated 12S, 16S, 18S, 28S and *cox*3. Colored boxes indicate clades defined in the text; the name of each clade is next to the colored box. Species epithets are given only for genera represented by more than one species; for a complete list of taxa, see Table S1. Numbers above the branches are bootstrap resampling values (parsimony [TNT] and ML, respectively) expressed as a percent followed by posterior probabilities (multiplied by100 for legibility); values <50 indicated by “–”; filled-in circles indicate nodes with support of 100% for all inferences.

Many relationships within Actiniaria, and between Actiniaria and other lineages of Hexacorallia are weakly supported, even when all sequence data are considered simultaneously (see Figs. 1, 2, generated using ML on the concatenated dataset without including SS guided alignments). Although a total evidence approach was taken (concatenating all five molecular markers) [46], a number of nodes within the phylogenetic reconstructions presented in Figs. 1 and 2 are not well supported, rendering some relationships among groups ambiguous or tentative. However, it is our interpretation that morphological features are consistent with the molecular results to such a degree that we can confidently discuss these newly-revealed relationships and proceed with erecting a new higher-level classification based on our current results with the caveat that future analyses should incorporate more variable markers to confirm or challenge the results presented here.

### Phylogeny of Hexacorallia and Actiniaria

All methods of phylogenetic inference (Parsimony [TNT & POY], ML [RAxML], Bayesian [MrBayes & PhyloBayes], and inferred SS) agreed in basic topology for the reduced concatenated dataset. In the resulting phylogenetic reconstruction the order Actiniaria is not monophyletic–*Boloceroides daphneae*, previously placed within the infraorder Boloceroidaria, is resolved outside of Actiniaria as sister to the clade containing members of order Zoanthidea (Fig. 1). All other members of Actiniaria cluster into two clades: Endocoelantheae+Edwardsiidae and a large and heterogeneous group that encompasses most of what Carlgren [12] considered Nynantheae. None of the subordinal groups proposed by Carlgren are monophyletic, and their relationships do not align with their ranking or hierarchical position in Carlgren’s [12] system (Table 1).

**Table 1:** Relationships and interpretation of higher-level taxa in Carlgren’s [12] classification.

Taxon name			Phylogenetic result
Protantheae			Sister to Boloceroidaria
Ptychodacteae			Polyphyletic because its members are not recovered as sister taxa;clustered with members of former Endomyaria
Endocoelantheae			Sister to athenarian family Edwardsiidae; together these clades arere-classified as suborder Anenthemonae
Nynantheae			Polyphyletic because of the relationship between Edwardsiidae and Endocoelantheae andbecause members of Protantheae and Ptychodacteae are recovered as sister to its members
	Boloceroidaria		*Boloceroides mcmurrichi* and *Bunodeopsis* nested among acontiate taxa;*B. daphneae* apart from other Actiniaria (see text)
	Athenaria		Polyphyletic: families formerly in this suborder distributed across tree assister to former members of Endomyaria, Acontiaria, and Endocoelantheae
	Thenaria		Boloceroidaria, Protantheae, Ptychodacteae, and most Athenaria nest within this group
		Endomyaria	Paraphyletic: includes Pychodacteae and some Athenaria
		Mesomyaria	Polyphyletic: one clade at base of Nynantheae, other lineages areassociated with former members of Acontiaria
		Acontiaria	Paraphyletic; includes several lineages formerly in Mesomyariaand Athenaria, plus Boloceroidaria and Protantheae

Carlgren [12] considered Ptychodacteae an order; Cappola and Fautin [15] re-classified this as a suborder. See [11] for extensive discussion of the composition and classification of Mesomyaria and Acontiaria.

We find a monophyletic Hexacorallia and monophyly of all orders within it, except Actiniaria (Fig. 1). Monophyly of Hexacorallia, Antipatharia, Ceriantharia, Corallimorpharia, Scleractinia, and Zoanthidea are each well supported; of these, only one of the least-well sampled taxon, Corallimorpharia, has bootstrap support for monophyly <100%. The clade that includes all Actiniaria except *Boloceroides daphneae* has bootstrap support of 100% in the concatenated ML analysis (Fig. 1).

The relationships among ordinal groups supported by our ML analysis of the concatenated dataset (Fig. 1) broadly follow those recovered by Daly et al. [7]. Ceriantharia is sister to all other lineages of Hexacorallia. Within the crown Hexacorallia clade, Actiniaria (exclusive of *Boloceroides daphneae*) is sister to all other ordinal-level lineages. Scleractinia and Corallimorpharia group together in a well-supported, monophyletic clade; this clade is sister to a clade containing Antipatharia, Zoanthidea, and *B. daphneae*.

Relationships among Actiniaria vary most significantly in terms of relationships within the large clade of former members of Carlgren’s [12] Nynantheae; the clade that includes Endocoelantheae and Edwardsiidae is well supported and recovered in almost all analyses (Table 2). We find a clade that broadly corresponds to the Endomyaria of Carlgren [12]; however, the position of *Epiactis* and *Capnea* renders the clade as paraphyletic. This clade includes some former members of Athenaria (e.g., *Harenactis, Stephanthus*, *Haloclava*, *Peachia*) and members of Ptychodacteae (e.g., *Dactylanthus*, *Preactis*) (Fig. 2). The ptychodactean species are not resolved as sister taxa. This large clade of endomyarians, athenarians, and ptychodacteans is sister to a clade corresponding to Rodríguez et al.’s [11] Metridioidea (Fig. 2). We find low support values for Metridioidea and its sublineages (except for MrBayes); as is expected given the expansion of the taxon sample, the support values are generally lower than in [11]. The protantheans *Protanthea* and *Gonactina* lie within Metridioidea, in a well-supported sister relationship to the boloceroidarians *Boloceroides mcmurrichi* [47] and *Bunodeopsis*. No members of Endocoelantheae or Protantheae have previously been included in a phylogenetic analysis.

**Table 2:** Representation of conflicts in topologies among markers and phylogenetic inferences.

Clade	Parsimony (TNT)								Parsimony (POY)							
	12S	16S	18S	28S	*cox*3	mt	nc	conct	12S	16S	18S	28S	*cox*3	mt	nc	conct
**Anenthemonae**	D	A	A	A	A	A	A	A	A	A	A	A	A	X	A	A
**Edwardsidioidea**	D	A	A	A	A	A	A	A	A	A	A	A	A	A	A	A
**Actinernoidea**	D	A	A	A	A	A	A	A	A	A	A	A	A	X	A	A
**Enthemonae**	U	**X**	A	A	A	A	A	A	A	A	A	A	A	A	A	A
**Actinostolina**	D	A	U	A	A	D	U	A	D	X	X	D	A	A	X	A
**Actinoidea**	D	D	U	A	A	D	U	A	D	D	X	A	X	A	A	A
Metridioidea	U	**X**	U	A	A	D	U	A	A	D	X	D	A	A	D	A
Boloceroidaria	D	D	D	A	A	A	A	A	A	A	X	A	A	A	A	A
Protantheae	D	D	D	A	A	A	A	A	A	D	A	A	A	A	A	A
Ptychodacteae	A	A	D	A	A	A	A	A	A	A	A	A	A	A	A	A
***Relicanthus daphneae***	D	D	D	D	D	A	D	D	D	D	D	D	A	A	D	A
	**ML**								**SS**							
	**12S**	**16S**	**18S**	**28S**	***cox*** **3**	**mt**	**nc**	**conct**	**12S**	**16S**	**18S**	**28S**	***cox*** **3**	**mt**	**nc**	**conct**
**Anenthemonae**	A	A	A	A	A	A	A	A	A	-	A	A	-	-	-	A
**Edwardsidioidea**	A	A	A	A	A	A	A	A	A	-	A	A	-	-	-	A
**Actinernoidea**	A	A	A	A	A	A	A	A	A	-	U	A	-	-	-	A
**Enthemonae**	A	A	A	A	A	A	A	A	A	-	A	A	-	-	-	A
**Actinostolina**	A	A	X	A	A	D	A	A	D	-	X	A	-	-	-	A
**Actinoidea**	A	D	D	A	A	D	A	A	D	-	A	A	-	-	-	A
Metridioidea	A	X	D	A	A	D	A	A	D	-	D	A	-	-	-	A
Boloceroidaria	A	D	X	A	A	A	A	A	A	-	A	A	-	-	-	A
Protantheae	A	A	A	A	A	A	A	A	A	-	A	A	-	-	-	A
Ptychodacteae	A	X	A	A	A	A	A	A	A	-	A	A	-	-	-	A
***Relicanthus*** ** ***daphneae***	D	D	D	D	D	D	D	A	D	-	D	A	-	-	-	A
	**MrBayes**								**Phylobayes**							
	**12S**	**16S**	**18S**	**28S**	***cox*** **3**	**mt**	**nc**	**conct**	**12S**	**16S**	**18S**	**28S**	***cox*** **3**	**mt**	**nc**	**conct**
**Anenthemonae**	A	A	A	A	A	A	A	A	A	A	-	A	U	-	-	-
**Edwardsidioidea**	A	A	A	A	A	A	A	A	A	A	-	A	A	-	-	-
**Actinernoidea**	A	A	A	A	A	A	A	A	A	A	-	A	A	-	-	-
**Enthemonae**	A	A	A	A	A	A	A	A	A	A	-	A	U	-	-	-
**Actinostolina**	D	A	X	A	A	A	A	A	D	A	-	A	A	-	-	-
**Actinoidea**	D	D	D	A	A	A	A	A	D	D	-	A	A	-	-	-
Metridioidea	D	X	D	A	A	A	A	A	D	X	-	A	A	-	-	-
Boloceroidaria	A	D	A	A	A	A	A	A	A	D	-	A	A	-	-	-
Protantheae	A	D	A	A	A	A	A	A	A	A	-	A	A	-	-	-
Ptychodacteae	A	X	A	A	A	A	A	A	A	A	-	A	A	-	-	-
***Relicanthus*** ** ***daphneae***	D	D	A	D	U	D	A	A	D	A	-	D	D	-	-	-

Comparisons made using results from ML of the concatenated dataset as reference; A, agree; U, unresolved; D, disagree in placement but agree on monophyly; X, disagree in placement and monophyly. Relationships among groups are considered at the higher-level (i.e., Ptychodacteae is considered as a group although is never recovered monophyletic, see text). Conct, concatenated; (−), data not available (SS alignment only available for 12S, 18S and 28S; some datasets did not converge in Phylobayes). Newly-erected higher-level groups in bold.

### Conflict among Methods of Phylogenetic Inference and Molecular Markers

Although the general results of ordinal monophyly and relationship are common to most methods and parameter sets, some individual data sets and analyses revealed alternative relationships. We have represented these conflicts in Table 2 using as reference for comparisons the results obtained for the concatenated dataset under ML. Under selected parameter costs (Table S6), POY recovered *Boloceroides daphneae* as sister to Actiniaria, a result that confirms the unique genetic signature of *B. daphneae* but also suggests that this taxon still belongs to Actiniaria.

Results based on single markers showed less congruence among methods or across parameter sets (Table 2). Tree topologies resulting from analyses of mitochondrial and nuclear markers differed across most methods of inference. For example, in parsimony analyses, Actinostolina was either sister to Endomyaria (mtDNA) or unresolved within a larger clade that includes Endomyaria+Metridioidea (ncDNA); whereas ML analyses resolved Actinostolina as sister to Endomyaria (mtDNA) or sister to Endomyaria+Metridioidea (ncDNA). MrBayes resolved Actinostolina as sister/basal to Endomyaria+Metridioidea using both mt- and nc-DNA. In other cases (ML and MrBayes) topological disagreement occurred at more critical nodes: mitochondrial genes show *Boloceroides daphneae* as sister to Actiniaria and cerianthids nested within Actiniaria, whereas nuclear genes resolved *B. daphneae* outside Actiniaria (usually sister to zoanthids with relatively low support (62%)) and Ceriantharia as sister to all other hexacorals. Parsimony analyses using TNT never recovered *B. daphneae* as sister to Actiniaria but always found Ceriantharia as sister to the remaining hexacorals. Searches of mitochondrial genes with POY resolved Antipatharia and Ceriantharia within Actiniaria, whereas nuclear genes analyzed with POY resolved Ceriantharia sister to the remaining hexacorallian orders. Furthermore, we found considerable variation in branch lengths among individual markers (results not shown, conflicts summarized in Table 2). Most disagreements can be attributed to a lack of data coverage; for example, *cox*3 recovered cerianthids within Actiniaria; however, coverage for this gene was incomplete in outgroups (see Table S1). Mitochondrial 16S resolved the former suborder Protantheae as sister to the remaining Actiniaria rather than nested within Metridioidea and sister to Boloceroidaria; however, 16S sequence data could not be obtained for *B. mcmurrichi*.

Of particular interest was the inability of both Bayesian inference methods–PhyloBayes and MrBayes–to converge on an optimal solution across a number of datasets (even after we modified a number of parameters), a result we attribute to either a lack of informative variability in our current set of markers or conflict among the different markers when concatenated.

### Classification and Taxonomic Results

In accordance with our results, we propose a new higher-level classification for the order Actiniaria, and adjust the taxonomic status of *Boloceroides daphneae*. We recognize two suborders within Actiniaria, Anenthemonae and Enthemonae. Anenthemonae includes members of Edwardsiidae and the former suborder Endocoelantheae; in members of this lineage mesenteries are not arranged in the typical paired, coupled arrangement seen in other actiniarians and in e.g. Corallimorpharia and Scleractinia. Anenthemonae contains two superfamilies: Edwardsioidea (members of the family Edwardsiidae, which have only eight perfect mesenteries) and Actinernoidea (members of the former suborder Endocoelantheae, which secondary cycles of mesenteries arise in the endocoels). Enthemonae includes the majority of actiniarians (members of former suborders Protantheae, Ptychodacteae, and Nynantheae and subgroups within), all of which have the typical arrangement of mesenteries for actiniarians (primarily hexamerous cycles in which pairs of mesenteries arise in the exocoels). Enthemonae contains three clades that we rank as superfamilies: Actinostoloidea (members of former family Actinostolidae *sensu* [10]), Actinoidea (members of former Endomyaria and Ptychodacteae plus some former members of Athenaria), and Metridioidea (acontiate actiniarians plus several families that have lost acontia, including members of former sub- and infra-orders Protantheae and Boloceroidaria, respectively). Diagnostic features and constituents of each group are listed in Appendix 1.

**Figure 3:**
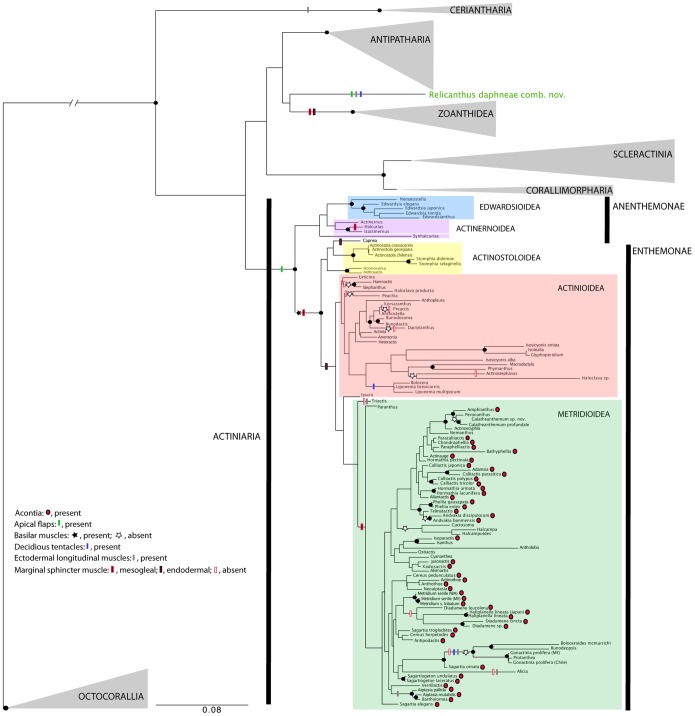
Ancestral character state reconstruction of morphological characters within Hexacorallia. Representation of ancestral character state reconstruction for seven morphological characters within Hexacorallia (acontia, basilar muscles, longitudinal ectodermal muscles, deciduous tentacles, marginal sphincter muscle, microorganism endosymbiosis, apical flaps). Characters mapped onto the Maximum Likelihood (ML) analysis of concatenated 12S, 16S, 18S, 28S and *cox*3. Characters absent unless specified in the figure. Directionality of acontia not inferred due to lack of support of nodes. Colored boxes indicate clades defined in the text; name of each clade is next to the colored box. Species epithets are given only for genera represented by more than one species; for a complete list of taxa, see Table S1. Only bootstrap resampling values (parsimony [TNT] and ML, respectively) >90% and values of posterior probabilities of 0.9 are indicated (by filled-in circles) for legibility; refer to Figures 1 and 2 for other support values.

Although its precise position is as yet unclear, no method or data set supports a close relationship between *Boloceroides daphneae* and the other members of Boloceroididae (Figs. 1, 3, Table 2). To address this situation, we create a new genus and family for this species. In light of the varied placement of this taxon in our analyses, we consider Relicanthidae fam. nov. Hexacorallia *incerti ordinis* and thus not within the classification scheme proposed above for Actiniaria.

**Relicanthidae fam. nov**., Rodríguez and Daly 2014.

Diagnosis. Solitary, skeleton-less hexacorallian with well-developed pedal disc but without basilar muscles. No marginal sphincter muscle. Longitudinal ectodermal muscles in column. Deciduous tentacles with sphincter at the base. Twenty-four pairs of perfect mesenteries arranged hexamerously. Muscles of mesenteries weak. Cnidom: Gracile spirocysts, basitrichs, microbasic *p-*mastigophores (*p*-rhabdoid A *sensu* [48]). Basitrichs with apical flaps.

Included genera. *Relicanthus* gen. nov, Rodríguez and Daly 2014 (for *Relicanthus daphneae* comb. nov.). The LSID identifier for this taxon is urn:lsid:zoobank.org:act:6611427D-6573-4452-B62C-97C1685EF853.

Etymology and nomenclature. Name comes from the Latin word “*reliquiae*” meaning “old remains.” *Relicanthus* gen. nov. is the type genus by monotypy.

**Figure 4:**
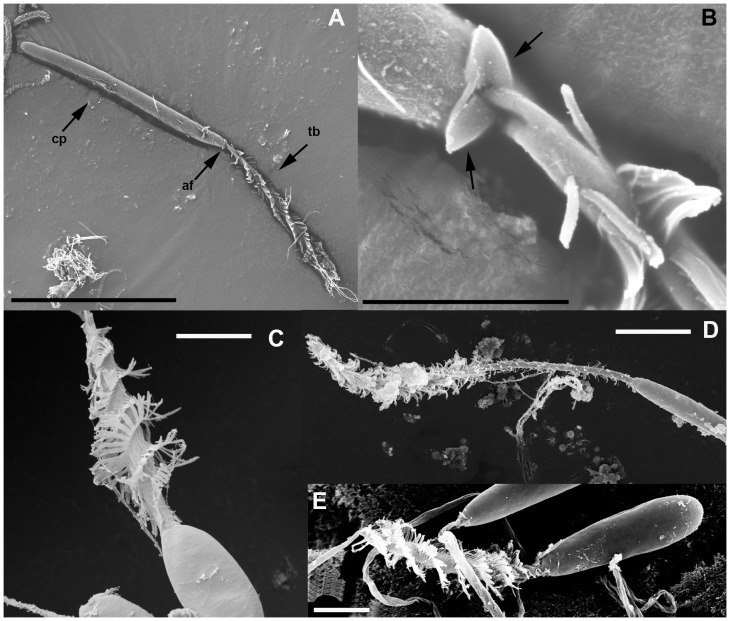
Cnidae capsules and apical flaps. A) SEM image of a discharged capsule of a basitrich from the tentacle of *Relicanthus daphneae* comb. nov. showing apical flaps. B) Detail of the apical flaps (arrows) of *R. daphneae* comb. nov. C) SEM image of a discharged capsule of a microbasic *p*-mastigophore from a member of Scleractinia; notice the absence of apical flaps. D) SEM image of a discharged capsule of a microbasic *p*-amastigophore from a member of Actiniaria (*Metridium*). E) SEM image of a discharged capsule of a microbasic *p*-mastigophore from a member of Actiniaria (*Bunodosoma*); notice differential disposition of larger spines between microbasic *p*-amastigophores (distal to capsule, E) and *p*-mastigophores (proximal to capsule, C, D). Abbreviations: af, Apical flaps; cp, Capsule; tb, tubule. Scale bars: A, 30 µm; B, E, 3 µm; C, 5 µm; D, 10 µm.

Remarks. Although molecular data do not closely affiliate this taxon with Actiniaria, the ultrastructure of its nematocysts strongly suggest that it belongs within Actiniaria: basitrichs of *Relicanthus daphneae* comb. nov. have apical flaps (Fig. 4) and the distal tubule of microbasic *p*-mastigophores of *R. daphneae* comb. nov. does not have spines; these features recall those of Actiniaria (see [1], [48], [49]).

***Relicanthus*** **gen. nov.** Rodríguez and Daly 2014.

Diagnosis (after [31]). Relicanthidae with adherent base. Column cylindrical, smooth, not divisible into regions. Tentacles numerous, extremely long and strongly tapering. Mesenteries hexamerously arranged in four cycles; two pairs of directives attached to single weak siphonoglyph. Longitudinal muscles of tentacles and radial muscles of oral disc ectodermal. Muscles of mesenteries weak. Cnidom: gracile spirocysts, basitrichs, microbasic *p-*mastigophores.

Included species. *Relicanthus daphneae* comb. nov. The type species of the genus is *Boloceroides daphneae* [31] by monotypy. The LSID identifier for this taxon is urn:lsid:zoobank.org:act:3A774C26-1B0F-4FA6-AC90-16CCC2CE79D9.

## Discussion

### New Higher-level Classification and Monophyly of Actiniaria

Our findings highlight the inadequacy of the previous higher-level classification for the order Actiniaria (Table 1). Although the relationships among groups identified by Carlgren [12] do not accord with our phylogenetic results, several of the lineages he identified in his classification are monophyletic: Boloceroidaria, Endocoelantheae, and Protantheae are each monophyletic, but they nest within larger groups rather than representing lineages equivalent in the phylogenetic hierarchy of Actiniaria (Table 1, Figs. 2, 3).

Perhaps because of the simplicity of their body plan, actiniarians show high levels of morphological convergence, and many of the relatively few morphological characters of the order have been repeatedly lost. This also applies to the features highlighted in Carlgren’s [12] classification, which tend to be applicable at more general levels than he inferred. Ectodermal longitudinal muscles are characteristic of the clade that includes the protantheans and the boloceroidarians, not uniquely derived in each. Contra Carlgren [50], this is a derived rather than primitive condition in these lineages. Basilar muscles are characteristic of all enthemonaean anemones and are lost multiple times in each sublineage (Fig. 3). Although Carlgren’s system, like Stephenson’s [13] before it, implied that endodermal and mesogleal marginal muscles represent independent and alternative derivations of marginal musculature, the optimization of marginal sphincter muscles on our trees (Fig. 3) and a recent study of this feature in members of Anenthemonae [51] suggest that marginal musculature is characteristic of Enthemonae, and that they arose as mesogleal muscles, being transformed into an endodermal muscle in the lineage leading to Actinoidea. Marginal musculature has arisen at least three times within Hexacorallia, and in each case (Enthemonae, the anenthemonean *Halcurias*, and in Zoanthidea; see Fig. 3) it arose as mesogleal muscles.

Nonetheless, our findings based on DNA sequence data correspond neatly with several morphological trends observed in the order Actiniaria. The primary distinction among actiniarians (that defining the newly-erected suborders Anenthemonae and Enthemonae) corresponds with the arrangement of the mesenteries. In members of Anenthemonae, the mesenterial arrangement departs from the common hexacorallian arrangement of hexamerous cycles of pairs of mesenteries that arise in the exocoels (space between different pairs of mesenteries) [12]. Similarly, within Anenthemonae, the major split also corresponds to the arrangement of mesenteries: in Edwardsioidea the eight perfect mesenteries arise in exocoels whereas in Actinernoidea secondary pairs of mesenteries arise in the endocoels (space within mesenteries of a pair) (see Appendix 1). The position of Edwardsiidae has been controversial: although Carlgren considered them a family within Nynantheae, others assigned higher-level ranks (e.g., [2], [52], [53], reviewed in [54]).

Within Enthemonae, acontia and marginal musculature stand out as phylogenetically-consistent features (Fig. 3). These characters have been used in previous classifications (e.g., [12], [55]; reviewed in [28]); we find them to apply at levels other than those suggested by previous workers, and find that both the marginal sphincter muscle and acontia have been lost several times (Fig. 3). Marginal musculature is lost in Edwardsioidea, Actinoidea (e.g., *Dactylanthus*; *Preactis*+*Korsaranthus*), and within Metridioidea (e.g., Boloceroididae+Protantheae, *Halcampa*+*Halcampoides*). Many of these instances of loss are associated with a reduction in body size or a shift to an infaunal habitat [6]. The endodermal or mesogleal nature of the marginal musculature also stands out as phylogenetically-consistent feature: members of Actinoidea have endodermal (or no) marginal sphincter and members of Actinostoloidea and Metridioidea have a mesogleal (or no) marginal sphincter muscle. However, the position recovered for *Epiactis* and *Capnea* challenges this pattern. Because support values across the tree are relatively low in general and the representation in our analysis of members of Actinoidea is not extensive relative to the diversity of the clade, the position of *Epiactis* and *Capnea* is not definitive. Also, the absence of structures in the column of *Epiactis* might suggest a basal position of this taxon, which might explain is position. Nevertheless, other features, such as cnidae (see below), support the position of *Epiactis* and *Capnea* within Actinoidea. Acontia are inferred to have arisen once, at the base of Metridioidea, and to have been lost multiple times (e.g., Boloceroididae+Protantheae; *Halcampa*+*Halcampoides*; *Ostiactis*: see discussion in [11]). In the case of acontia, the position of *Triactis* and *Paranthus* challenges this generalization. Nevertheless, *Triactis* belongs to Aliciidae, a family not well sampled in this study (only two taxa included), which have many modifications to their anatomy (its members have lost the marginal musculature, gained unique column structures, etc.). As with *Epiactis* and *Capnea,* cnidae (see below) support the position of *Triactis* within Metridioidea. A denser taxon sampling is required to address the placement and support of these specific taxa.

Cnidae have been interpreted to provide the only synapomorphy for Actiniaria, as all Actiniaria (and only Actiniaria) have nematocysts with apical flaps and have no spines on the distal tubule of *p*-mastigophores [1], [48]. However, the phylogenetic position recovered in our analyses for *Relicanthus daphneae* comb. nov. and the presence of apical flaps and *p*-mastigophores without spines in the distal tubule in nematocysts of this taxon (Fig. 4) raise two possibilities: 1) these features of the cnidae are not a synapomorphy for sea anemones, or 2) the molecular data incorrectly place *R. daphneae* comb. nov. outside of Actiniaria. The origin and evolution of apical flaps remains unresolved until the position of *Relicanthus* within Hexacorallia is resolved with higher support. Similarly, the order Actiniaria remains characterized by the absence of attributes defining other orders of hexacorals. Nevertheless, although the current classification of cnidae is not useful for phylogenetic inference, cnidae have been shown to carry phylogenetic information, with different clades associated with different types of cnidae [56]. For example, *p*-rhabdoids B (∼microbasic *p*-amastigophores) characterize acontiate actiniarians (superfamily Metridioidea) whereas *p*-rhabdoids A (∼microbasic *p*-mastigophores) are common in the other clades (e.g., Actinoidea, Actinostoloidea, etc.) (Fig. 4). Our results confirm this: the cnidom of members of former Boloceroidaria and Protantheae resemble those of acontiate actiniarians in having *p*-rhabdoids B but no *p*-rhabdoids A. Similarly, Aliciidae, a family traditionally placed within Endomyaria [12], [14] but here recovered within Metridioidea, has a cnidom that includes *p*-rhabdoids B and lacks *p*-rhabdoids A, a finding that supports its new placement within Metridioidea.

Metridioidea includes several lineages that have lost or modified many of the features that characterize the group as a whole. Taxa belonging to what had previously been the sub- and infra-orders Protantheae and Boloceroidaria (respectively) form a clade of highly modified species in which several morphological characters (e.g., marginal sphincter and basilar muscles, etc.) have been lost or reduced. Within Metridioidea, we also find clades (e.g., Deepsina, Chemosynthina: [10], [11]) in which acontia have been lost, and others, such as Graspina in which the base is highly modified to exploit substrates in the deep-sea.

### A Case of Morphological Convergence, Boloceroidaria and *Relicanthus Daphneae* Comb. Nov

Molecular data strongly suggest that *Relicanthus daphneae* comb. nov. represents a lineage distinct from other Actiniaria. Despite the ambiguity about its precise placement within Hexacorallia, our results are conclusive about the lack of a close relationship between *Relicanthus daphneae* comb. nov. and other members of the former infraorder Boloceroidaria. Our results show an extreme case of morphological convergence between *Relicanthus* gen. nov. and the boloceroidarians. Like members of Boloceroididae, *R. daphneae* comb. nov. has ectodermal column muscles, autotomizes its tentacles, lacks a marginal sphincter muscle, and lacks basal musculature despite having a distinct pedal disc. Nonetheless, as noted in its original description, *R. daphneae* comb. nov. differs in ecology and size from more “typical” boloceroidids, which are generally small polyps (about 20 mm column length), live in shallow and warm waters, and usually show active swimming behavior to escape predators (reviewed in [57]). In contrast, *R. daphneae* comb. nov. is the largest actiniarian described to date (column to 1 m diameter), from an extreme deep-sea environment (hydrothermal vents), and shows no evidence of any swimming behavior or asexual reproduction (*R. daphneae* comb. nov. is gonochoric with the largest eggs reported, see [31]). Autotomy of tentacles is a common feature in other deep-sea taxa (e.g., *Bolocera*, *Liponema*, *Iosactis*, see [58]). The lack of sphincter and basilar musculature, and the ectodermal column musculature may arise by a common mechanism, as these features are characteristic of juvenile actiniarians, suggesting that paedomorphosis may have led to this striking convergence.

### Functional and Evolutionary Correlates of Morphological Attributes

In our phylogenetic reconstructions we recognize several clades whose members have similar habitats or ecologies. These trends hint at the pattern and nature of radiation within this group and provide context for interpreting the putative function of some morphological attributes. Because our sampling is biased towards deep and shallow water, with relatively fewer species from 60–1000 m, and because many of these clades have relatively low support, these findings bear further exploration. The most distinct examples of ecological clades are perhaps Edwardsioidea, Actinoidea, Actinostoloidea and, within Metridioidea, the acuticulate and cuticulate clades (Fig. 2).

All members of Edwardsiidae are burrowers; these animals live in soft sediment, with only the tentacle crown exposed (reviewed in [54], [59]). Like most burrowing anemones, edwardsiids are small and lack basal musculature. Burrowing undoubtedly poses some constraints in terms of distribution, but the group is nonetheless diverse and widespread, occurring in every ocean and at all depths [14], often with multiple species co-occurring at a site or in a region (e.g., [55], [60], [61]).

The superfamily Actinoidea is primarily comprised of shallow-water forms. Members of this clade typically have an endodermal marginal sphincter muscle that allows the distal column to close tightly, retaining water within the column. Many actinoideans have some kind of adhesive structure (vesicles, verrucae, etc.) on the column; these may provide desiccation relief, UV protection, or camouflage [62]. Within Actinoidea is a distinctive deep-sea clade (e.g., *Bolocera*, *Liponema*, presumably *Iosactis*) whose members have long deciduous tentacles and tend to have a short column [58].

Actinostolina (superfamily Actinostoloidea) is a deep-sea and polar clade, and includes relatively large animals with smooth columns that are often internal brooders. Internal brooding in marine invertebrates is often associated with extreme environments and although it potentially limits the distribution of the species, it increases survival of the offspring in such environments. Nonetheless, internal brooding is also common in some members of Actinoidea in shallow-water habitats (reviewed in [63]).

Metridioidea contains an acuticulate clade (Fig. 2) comprised of shallow-water anemones with smooth columns, numerous perfect mesenteries, cinclides (perforations in the body wall associated with expulsion of acontia), and microbasic *p*-amastigophores in the acontia. Within this acuticulate clade we find the former members of suborder Protantheae and infraorder Boloceroidaria as sister taxa. These taxa form a well-supported clade of small-sized, highly-derived actiniarians that have lost several morphological characters–marginal sphincter muscle, basilar muscles, acontia– but share a synapomorphy of having ectodermal longitudinal muscles at least in the distal column. Because of similarities in the cnidom, Schmidt [49] associated members of families Aiptasiidae and Aliciidae with the former Protantheae and Boloceroidaria; we find that Aliciidae is sister to the clade containing former members of Protantheae and Boloceroidaria (except *Relicanthus daphneae* comb. nov.). The reductions in musculature and the retention of juvenile features such as ectodermal column muscles may reflect paedomorphosis.

In contrast, the cuticulate clade within Metridioidea (“Clade II” *sensu* [11]) contains mostly deep-sea forms, whose thick columns bear cuticle and (usually) tubercles. Despite their large size, perfect mesenteries are not numerous in these animals. Within Cuticulata, we see several taxa that have reduced or lost acontia (Fig. 3). Among these are members of the clade Graspina (families Actinoscyphiidae, Amphianthidae, and Galatheanthemidae), whose members have a modified pedal disc that enables them to exploit alternative substrates (e.g., tubes of worms, octocorals, etc.), a distinctive advantage in the deep sea because rocky outcrops may be limited. Actiniarians from deep-sea chemosynthetic environments (clade Chemosynthina, see [10], [11]) also share the loss of acontia; but in this case cinclides are still usually present.

This phylogeny provides context for understanding the evolution of muscles, a feature previous authors (e.g., [12], [13], [49]) considered of primary importance in classifying actiniarians. We infer that basal muscles are primitively absent in Anenthemonae (Fig. 3). This means that, contra Daly et al. [6], basilar muscles have not been lost in the burrowing Edwardsiidae, although they do seem to be lost in other burrowing lineages (e.g., *Halcampa*+*Halcampoides*, *Andvakia*, *Haloclava*). Marginal sphincter muscles are reduced or lost in several burrowing forms (e.g., *Halcampa*+*Halcampoides*, *Andvakia*, *Actinostephanus*) and in several non-burrowing forms (e.g., Aliciidae, Boloceroidaria+Protantheae); these muscles are most commonly lost in forms that undergo an overall reduction in column diameter, a reduction that may occur in concert with or separately from the adoption of a burrowing habit.

Small body size does not correlate with relatively fewer mesenteries, at least in a few critical cases. For example, the cuticulate clade within Metridioidea includes relatively large-bodied animals, but these all have relatively few mesenteries, when compared to members of the acuticulate clade or to members of Actinoidea. Size is more constrained in burrowing forms than in those that attach: Edwardsiidae encompasses animals with less variation in size than does Actinernoidea or any of the clades within Enthemonae.

Associations with photosymbiotic microorganisms occur across the actiniarian tree (within Edwardsiidae, Actinoidea, and in both the cuticulate and acuticulate clades of Metridioidea). Given its distribution within each clade and the biases in our taxon sampling towards shallow-water species (and thus toward those taxa in which this trait is likely to occur), it is difficult to make broad inferences about the distribution of this trait except to note that the capacity to engage with symbionts is broadly distributed across Actiniaria and probably analogous across Hexacorallia (Fig. 3). Although symbiosis with metazoans is similarly broadly distributed, those relationships show clearer ecological or lineage-specific patterns. Symbiosis with scyphozoans and ctenophorans has arisen in two very distantly-related lineages of burrowing anemones (Edwardsiidae and Haloclavidae, see [64] and [65] for *Edwardsiella* and *Peachia,* respectively). Symbiosis with hermit crabs has arisen once within Actinoidea (in *Stylobates*: reviewed in [66]) and multiple times within Cuticulata (reviewed in [67]).

With respect to other Hexacorallia, Actiniaria is characterized by “absence” of traits: its members lack a skeleton, are solitary, and have only a marginal ring of tentacles. Labial tentacles (in addition to marginal tentacles) characterize Ceriantharia and are inferred to be a synapomorphy for that clade. The lack of skeleton of Actiniaria is primary rather than secondary; the topology of the ML concatenated data set suggests that the skeleton of corals and that of Antipatharia arose as an independent event in each of those clades. Similarly, we infer the solitary state of Actiniaria to be ancestral and shared with Ceriantharia; the shift to colonial life occurs in its sister clade (see discussion in [7]). The marginal sphincter muscle borne by many anemones has an analog among hexacorallians in Zoanthidea, but this seems to be an independent origin; within Actiniaria our analyses suggest that the marginal sphincter muscle has also evolved independently twice, once within Actinernoidea (in *Halcurias*) and once at the base of the Enthemonae; in both instances the marginal sphincter seems to have arisen as a mesogleal muscle (Fig. 3).

It is currently impossible to date the age of the split between the major lineages of Actiniaria: there are no fossils for Actiniaria that can be associated with any specific clade, and rates of molecular evolution are heterogeneous within the order (see branch lengths, Fig. 2 and [9]) and within the larger clade to which Actiniaria belongs (see [68]). Nevertheless, the broader phylogeny of Hexacorallia provides some perspective because Actiniaria is at least as old as the constituents of its sister lineage. Fossil-based estimates for skeletonized hexacorals place the group deep in the Paleozoic: 425 mya for Scleractinia [69] and 470 mya for Antipatharia [70] (but see [71] for discussion of fossil identity). Because the divergence between Actiniaria and other hexacorallians must precede the emergence of each of these sublineages, that split must be at least 470 mya.

## Appendix 1. Taxonomic changes

We list authorship for taxa above species and the taxonomic changes made in this work. Authorship of new names here contained should be attribute to Rodríguez and Daly. We use italics to indicate modifications to cited diagnoses and asterisks to indicate those taxa whose placement has changed; underlined taxa are included in molecular analyses (Fig.1).

### Order Actiniaria [72]

Diagnosis (after [12]). *Hexacorallia with skeleton-less, solitary polyps*, with proximal end either rounded, physa-like or with a more or less well-developed, flat pedal disc; without or with basilar muscles. Column smooth or provided with verrucae, tenaculi, vesicles, marginal *projections* or other specializations of variable structure, often divisible into different regions, sometimes with spirocysts and with nematocyst batteries, rarely with ectodermal muscles. Margin indistinct or distinct, sometimes separated from tentacles by a more or less developed fosse. Tentacles retractile or not, usually arranged hexamerously in alternating cycles but sometimes in radial series at least in the case of those communicating with endocoels, usually simple, more rarely knobbed at the apex or branched or provided with papillae; exceptionally absent. Sphincter absent or present, endodermal to mesogloeal. Oral disc usually circular, but sometimes drawn out into lobes of varying appearance. Actinopharynx usually with two siphonoglyphs, but from one to several. Siphonoglyphs usually connected with directive mesenteries; exceptionally the single siphonoglyph is more or less wholly separated from actinopharynx. Pairs of mesenteries usually arranged in cycles, usually *hexamerously* (6+6+12, etc.); variable number of perfect pairs. After first 6 pairs or later, subsequent mesenteries grow either (a) from pedal disc upwards, or (b) from oral disc downward, or (c) more or less simultaneously from limbus and margin. Retractor muscles variable in shape, from diffuse to circumscribed. Parietobasilar muscles more or less strong; elongate forms usually with a well differentiated parietal muscle together with parietal part of longitudinal mesenterial muscles. Ciliated tracts of filaments as a rule present. Acontia present or absent. *Gametogenic tissue* at similar level as filaments; with variable distribution. Cnidom: spirocysts, atrichs, basitrichs, holotrichs, microbasic *b*- and *p*-mastigophores, microbasic and macrobasic *p*-amastigophores (all types never simultaneously present in any single individual). *Nematocysts (except p-rhabdoids A) with apical flaps.*

### Suborder Anenthemonae

Diagnosis. *Actiniaria* with proximal end either rounded, physa-like or with a more or less well-developed, flat pedal disc; without basilar muscles. Column smooth, with nematocyst batteries or cuticle and tenaculi; divisible or not into different regions, without longitudinal ectodermal muscles. Tentacles simple, retractile or not, usually arranged hexamerously in alternating cycles or in cycles related to mesenterial arrangement. Marginal sphincter muscle usually absent, if present weak and mesogloeal. Oral disc usually circular, but sometimes drawn out into lobes of varying appearance. Actinopharynx with one or two siphonoglyphs. Pairs of mesenteries distinctly arranged; either only 8 macrocnemes mesenteries and at least 4 microcnemes or in cycles with pairs of mesenteries after first 12 mesenteries (six couples), appearing in lateral endocoels with longitudinal muscles oriented as in directives. Retractor muscles variable in shape, from diffuse to circumscribed. Parietobasilar muscles more or less strong; elongate forms usually with a well differentiated parietal muscle together with parietal part of longitudinal mesenterial muscles. Ciliated tracts of filaments present. Acontia absent. Cnidom: spirocysts, atrichs, basitrichs, holotrichs, microbasic *b*- and *p*-mastigophores.

Included families. Edwardsiidae* [73]; Actinernidae* [13]; Halcuriidae* [74].

Etymology. Name comes from the Greek prefix “*an*-” meaning “not”, the Greek word “*Enthe*” meaning “groups” and the word anemone.

Remarks. This suborder includes actiniarians with unique arrangement of mesenteries, those departing from the most typical hexamerous cycles with pairs of mesenteries arising in exocoels (members of Edwardsiidae and the former suborder Endocoelantheae).

#### Superfamily Edwardsioidea [73]

Diagnosis (after [12]). Anenthemonae with elongate, vermiform body usually divisible into at least two regions, long scapus with cuticle and distal scapulus but often also with thin capitulum below tentacles. Aboral end rounded, naked physa. No marginal sphincter muscle or acontia. Only 8 macrocnemes mesenteries and at least 4 microcnemes. Macrocnemes divided into two pairs of directives and four lateral mesenteries, two on each side, with retractors facing ventral directives. Retractors diffuse to strongly restricted. Parietal muscles always distinct. Cnidom: spirocysts, atrichs, basitrichs, microbasic *p*-mastigophores.

Included families. Edwardsiidae*.

Remarks. We transfer Edwardsiidae (formerly included within the subtribe Athenaria) within suborder Anenthemonae.

#### Superfamily Actinernoidea [13]

Diagnosis (after [12], [51]). *Anenthemonae* with well-developed pedal disc but without basilar muscles. Column *smooth, or with nematocyst batteries*, nearly always with spirocysts. Margin tentaculate. Sphincter absent or weak mesogleal. Tentacles in variable number, often with thickened aboral side, either in two alternating cycles or although usually arranged in cycles, in peculiar way related to development of mesenteries. Longitudinal muscles of tentacles and radial muscles of oral disc ectodermal, with a slight mesogloeal tendency. Oral disc sometimes lobed. One or two siphonoglyphs. Usually more mesenteries than directives attached to siphonoglyphs. Unique mesenterial arrangement: after first 12 mesenteries (six couples) are developed, all subsequent pairs appear in lateral endocoels with longitudinal muscles oriented as in directives. Cnidom: spirocysts, basitrichs, holotrichs, microbasic *p*-mastigophores.

Included families. Actinernidae*; Halcuriidae*.

Remarks. We transfer members of the former suborder Endocoelantheae within newly-erected suborder Anenthemonae.

### Suborder Enthemonae

Diagnosis. Actiniaria with a rounded or flat aboral end with or without basilar muscles. Column smooth or with outgrowths, rarely (and then especially in distalmost part) with longitudinal ectodermal muscles. Sphincter absent or present, endodermal or mesogloeal. Tentacles simple or complex, commonly arranged in cycles, sometimes in radial rows. Siphonoglyphs usually attached to directives, rarely to non-directives, when directives are absent. Mesenteries as a rule arranged in cycles, commonly hexamerously. Secondary mesenteries always develop in exocoels. Pairs of non-directives consist of two mesenteries with retractors facing one another, rarely unpaired mesenteries occur. Mesenterial filaments with or without ciliated tracts. Cnidom: spirocysts, basitrichs, holotrichs, macrobasic *p*-mastigophores and p-amastigophores, microbasic *b*- and *p*-mastigophores and *p*-amastigophores.

Included families. All families previously included within Actiniaria (except Edwardisiidae, Actinernidae and Halcuriidae): Acontiophoridae [75]; Actiniidae [76]; Actinodendridae [77]; Actinoscyphiidae [78]; Actinostolidae [79]; Aiptasiidae [80]; Aiptasiomorphidae [12]; Aliciidae* [81]; Andresiidae* [13]; Andvakiidae [82]; Antipodactinidae [83]; Amphianthidae [72]; Bathyphelliidae [79]; Boloceroididae* [50]; Capneidae [84]; Condylanthidae [13]; Diadumenidae [78]; Exocoelactinidae* [85]; Gonactiniidae* [86]; Halcampidae [87]; Haliactinidae [12]; Haliplanellidae [88]; Haloclavidae* [89]; Hormathiidae [79]; Homostichanthidae [90]; Iosactinidae [91]; Isanthidae [75]; Kadosactinidae [92]; Limnactiniidae* [60]; Liponematidae [72]; Metridiidae [86]; Mimetridiidae [93]; Minyadidae [94]; Nemanthidae [95]; Nevadneidae* [96]; Octineonidae [97]; Oractinidae [98]; Ostiactinidae [11]; Phelliidae [99]; Phymanthidae [87]; Preactiniidae* [100]; Ptychodactinidae* [101]; Ramireziidae [93]; Sagartiidae [102]; Sagartiomorphidae [103]; Spongiactinidae [104]; Stichodactylidae [87]; Thalassianthidae [105].

Etymology. Name comes from the Greek word “*Enthe*” meaning “groups” and the word anemone.

Remarks. Members of this newly-erected suborder have mostly hexamerous cycles in which pairs of mesenteries arise in the exocoels.

#### Superfamily Actinostoloidea [79]

Diagnosis (after [12]). Enthemonae with basilar muscles, mesogleal marginal sphincter and no acontia or acontioids. Aboral end flattened and adherent. Column usually smooth. Tentacles and mesenteries usually numerous. Mesenteries not differentiated into macro- and microcnemes. Mesenteries of same pair often unequally developed. Retractors usually diffuse weak or strong, never circumscribed. Cnidom: gracile spirocysts, basitrichs, holotrichs, microbasic *b*- and *p*-mastigophores.

Included families. Actinostolidae; Exocoelactinidae*.

Remarks. Actinostoloidea includes members of former family Actinostolidae *sensu* [16]. In addition, we place the family Exocoelactinidae within Actinostoloidea until members of the family are available for molecular analysis based on its traditional placement within the former Mesomyaria [12].

#### Superfamily Actinioidea [76]

Diagnosis (after [12]). Enthemonae *as a rule with basilar muscles and endodermal or no marginal sphincter*. Aboral end flattened and usually adherent, distinctly differentiated from the column. Column of variable appearance, sometimes divisible into different regions; often with verrucae, marginal spherules or pseudospherules, vesicles or other protuberances. Tentacles and mesenteries usually numerous, the former cyclically or radially arranged. Mesenteries rarely differentiated into macro- and microcnemes. Retractors weak or strong, rarely circumscribed. Acontia absent. Cnidom: *gracile* spirocysts, basitrichs, *holotrichs*, microbasic *b*- and *p*-mastigophores, and macrobasic *p*-mastigophores.

Included families. Actiniidae; Actinodendridae; Andresiidae*; Capneidae; Condylanthidae; Haloclavidae*; Homostichanthidae; Iosactinidae; Limnactiniidae*; Liponematidae; Minyadidae; Oractinidae; Phymanthidae; Preactiniidae*; Ptychodactinidae*; Stichodactylidae; Thalassianthidae.

Remarks. The superfamily Actinioidea includes members of former Endomyaria plus several former athenarian families whose members are consistently recovered within this superfamily.

#### Superfamily Metridioidea [86]

Diagnosis (after [12] and [11]). *Enthemonae* as a rule with basilar muscles, mesogleal marginal sphincter and acontia or acontioids; in a few instances these characters may be absent or highly modified. Cnidom: robust and gracile spirocysts, basitrichs, holotrichs, microbasic *b*- and *p*-mastigophores and *p*-amastigophores and macrobasic *p*-amastigophores.

Included families. Acontiophoridae; Actinoscyphiidae; Aiptasiidae; Aiptasiomorphidae; Aliciidae*; Amphianthidae; Andvakiidae; Antipodactinidae; Bathyphelliidae; Boloceroididae*; Diadumenidae; Gonactiniidae*; Halcampidae; Haliactinidae; Haliplanellidae; Hormathiidae; Isanthidae; Kadosactinidae; Metridiidae; Mimetridiidae; Nemanthidae; Nevadneidae*; Octineonidae; Ostiactinidae; Phelliidae; Ramireziidae; Sagartiidae; Sagartiomorphidae.

Remarks. We transfer the families Boloceroididae, Nevadneidae, and Gonactiniidae to Metridioidea. Although they lack acontia, they are consistently recovered among acontiate taxa, and thus are re-classified as belonging to this superfamily, rather than to Protantheae and Boloceroidaria, respectively. We also transfer Aliciidae to Metridioidea rather than include it within Actinoidea for similar reasons. Furthermore, cnidae data support all these new placements.

## Supporting Information

Table S1 **Taxa included in this study, with voucher location and accession numbers.**
(DOCX)

Table S2 **Parameters implemented within MrBayes v.3.1.2.**
(DOCX)

Table S3 **Parameters implemented in MrBayes v.3.1.2.**
(DOCX)

Table S4 **Parameters implemented in PhyloBayes v3.3b.**
(DOCX)

Table S5 **Ancestral state reconstruction of seven morphological characters.**
(DOCX)

Table S6 **Tree statistics for the sensitivity analysis for the different parameter set values of POY analyses.**
(DOCX)

Text S1 **Details of material and methods.**
(DOCX)
