# Supplementary material for: Hidden among Sea Anemones: The First Comprehensive Phylogenetic Reconstruction of the Order Actiniaria (Cnidaria, Anthozoa, Hexacorallia) Reveals a Novel Group of Hexacorals
Source: PLoS One. 2014 May 7;9(5):e96998. doi: 10.1371/journal.pone.0096998 (PMC4013120; doi:10.1371/journal.pone.0096998)
Supplement: Table S1 — Taxa included in this study, with voucher location and accession numbers. (DOCX) [file pone.0096998.s001.docx]

**Table S1. Taxa included in this study, with voucher location and accession numbers.**

| **Higher Taxon** | **Family** | **Genus** | **Species** | **Voucher** | **12S** | **16S** | **18S** | **28S** | ***Cox*3** |
| --- | --- | --- | --- | --- | --- | --- | --- | --- | --- |
| Actiniaria |  |  |  |  |  |  |  |  |  |
| **Actinernoidea** | **Actinernidae** | *Actinernus* | *antarcticus* | AMNH | *KJ482930* | *KJ482966* | *KJ483023* | *KJ483126* | ------------ |
|  |  | *Isactinernus* | *quadrolobatus* | AMNH | *KJ482932* | *KJ482968* | *KJ483024* | *KJ483105* | *KJ482998* |
|  |  | *Synhalcurias* | *laevis* | NA | *KJ482942* | *------------* | *KJ483021* | *KJ483120* | ------------- |
|  | **Halcuriidae** | *Halcurias* | *pilatus* | AMNH | *KJ482931* | *KJ482967* | *KJ483020* | *KJ483109* | *KJ482997* |
| **Actinoidea** | Actiniidae | *Actinia* | *fragacea* | CAS | EU190714 | EU190756 | EU190845 | *KJ483085* | GU473334 |
|  |  | *Anemonia* | *viridis* | CAS | EU190718 | EU190760 | EU190849 | *KJ483095* | GU473335 |
|  |  | *Anthopleura* | *elegantissima* | KUNHM | EU190713 | EU190755 | EU190844 | *KJ483104* | GU473333 |
|  |  | *Anthostella* | *stephensoni* | AMNH | JQ810719 | JQ810721 | JQ810723 | *KJ483132* | JQ810726 |
|  |  | *Bolocera* | *kerguelensis* | AMNH | *KJ482925* | *KJ482965* | *KJ483029* | *KJ483133* | *KJ482985* |
|  |  | *Bunodactis* | *verrucosa* | KUNHM | EU190723 | EU190766 | EU190854 | *KJ483084* | ------------- |
|  |  | *Bunodosoma* | *grandis* | KUNHM | EU190722 | EU190765 | EU190853 | *KJ483083* | GU473336 |
|  |  | *Epiactis* | *lisbethae* | KUNHM | EU190727 | EU190771 | EU190858 | EU190816 | GU473360 |
|  |  | *Glyphoperidium* | *bursa* | AMNH | *KJ482923* | *KJ482961* | *KJ483033* | *KJ483136* | *KJ482982* |
|  |  | *Isotealia* | *antarctica* | AMNH | JQ810720 | JQ810722 | ----------- | ----------- | JQ810727 |
|  |  | *Isosicyonis* | *alba* | AMNH | ----------- | *KJ482959* | *KJ483030* | *KJ483134* | *KJ482981* |
|  |  | *Isosicyonis* | *striata* | AMNH | EU190736 | EU190781 | EU190864 | *KJ483137* | FJ489493 |
|  |  | *Korsaranthus* | *natalinesis* | AMNH | *KJ482920* | *KJ482958* | *KJ483017* | *KJ483117* | *KJ482987* |
|  |  | *Macrodactyla* | *doreenensis* | KUNHM | EU190739 | EU190785 | EU190867 | *KJ483049* | GU473342 |
|  |  | *Urticina* | *coriacea* | KUNHM | GU473282 | EU190797 | EU190877 | *KJ483094* | GU473351 |
|  | Actinodendridae | *Actinostephanus* | *haeckeli* | KUNHM | *KJ482936* | EU190762 | *KJ483034* | ----------- | GU473353 |
|  | Capneidae | *Capnea* | *georgiana* | AMNH | ----------- | *KJ482951* | *KJ483022* | *KJ483050* | *KJ482990* |
|  | **Haloclavidae** | *Haloclava* | *producta* | KUNHM | EU190734 | EU190779 | AF254370 | *KJ483097* | JF833008 |
|  |  | *Haloclava* | sp. | AMNH | *KJ482924* | *KJ482963* | *KJ483031* | *KJ483138* | *KJ482989* |
|  |  | *Harenactis* | *argentina* | AMNH | *KJ482926* | *KJ482964* | *KJ483026* | *KJ483047* | *KJ482984* |
|  |  | *Peachia* | *cylindrica* | KUNHM | EU190743 | EU190789 | *KJ483015* | EU190732 | ------------ |
|  |  | *Stephanthus* | *antarcticus* | AMNH | *KJ482927* | *KJ482960* | *KJ483019* | *KJ483092* | *KJ482983* |
|  | Liponematidae | *Liponema* | *brevicornis* | KUNHM | EU190738 | EU190784 | EU190866 | *KJ483139* | *KJ483001* |
|  |  | *Liponema* | *multiporum* | AMNH | *KJ482922* | *KJ482962* | ----------- | ----------- | ----------- |
|  | Phymanthidae | *Phymanthus* | *loligo* | KUNHM | EU190745 | EU190791 | EU190871 | ----------- | GU473345 |
|  | **Preactiidae** | *Dactylanthus* | *antarcticus* | AMNH | GU473272 | AY345877 | AF052896 | *KJ483086* | GU473358 |
|  |  | *Preactis* | *milliardae* | AMNH | *KJ482921* | *KJ482957* | *KJ483018* | *KJ483118* | *KJ482986* |
|  | Stichodactidae | *Heteractis* | *magnifica* | KUNHM | EU190732 | EU190777 | EU190862 | *KJ483093* | *KJ482988* |
| **Actinostoloidea** | Actinostolidae | *Actinostola* | *crassicornis* | AMNH | ----------- | EU190753 | EU190843 | *KJ483098* | GU473332 |
|  |  | *Actinostola* | *chilensis* | AMNH | ----------- | GU473285 | GU473302 | *KJ483110* | GU473357 |
|  |  | *Actinostola* | *georgiana* | AMNH | *KJ482928* | *KJ482952* | *KJ483032* | *KJ483099* | *KJ482991* |
|  |  | *Antholoba* | *achates* | AMNH | GU473269 | GU473284 | GU473301 | *KJ483128* | GU473356 |
|  |  | *Anthosactis* | *janmayeni* | AMNH | *KJ482938* | GU473292 | GU473308 | *KJ483091* | GU473363 |
|  |  | *Hormosoma* | *scotti* | AMNH | EU190733 | EU190778 | EU190863 | *KJ483090* | GU473366 |
|  |  | *Paranthus* | *niveus* | AMNH | GU473277 | GU473295 | GU473311 | *KJ483072* | GU473344 |
|  |  | *Stomphia* | *didemon* | KUNHM | *KJ482929* | EU190795 | EU190875 | *KJ483127* | GU473348 |
|  |  | *Stomphia* | *selaginella* | AMNH | GU473280 | GU473298 | GU473314 | GU473331 | GU473349 |
| **Edwardsioidea** | **Edwardsiidae** | *Edwardsia* | *elegans* | AMNH | EU190726 | EU190770 | EU190857 | *KJ483087* | GU473338 |
|  |  | *Edwardsia* | *japonica* | KUNHM | GU473274 | GU473288 | GU473304 | *KJ483048* | GU473359 |
|  |  | *Edwardsia* | *timida* | KUNHM | GU473281 | ------------- | GU473315 | *KJ483088* | *KJ482996* |
|  |  | *Edwardsianthus* | *gilbertensis* | AMNH | EU190728 | EU190772 | EU190859 | EU190817 | ------------- |
|  |  | *Nematostella* | *vectensis* | KUNHM | EU190750 | AY169370 | AF254382 | *KJ483089* | FJ489501 |
| Metridioidea | Actinoscyphiidae | *Actinoscyphia* | *plebeia* | AMNH | EU190712 | EU190754 | FJ489437 | *KJ483067* | FJ489476 |
|  | Aiptasiidae | *Aiptasia* | *mutabilis* | KUNHM | JF832963 | FJ489418 | FJ489438 | *KJ483115* | FJ489505 |
|  |  | *Aiptasia* | *pallida** | KUNHM | EU190715 | EU190757 | EU190846 | EU190803 | *KJ482979* |
|  |  | *Bartholomea* | *annulata* | AMNH | EU190721 | EU190763 | EU190851 | *KJ483068* | FJ489483 |
|  |  | *Neoaiptasia* | *morbilla* | KUNHM | EU190742 | EU190788 | EU190869 | *KJ483075* | JF833010 |
|  | **Aliciidae** | *Alicia* | *sansibarensis* | AMNH | *KJ482933* | *KJ482953* | *KJ483016* | *KJ483116* | *KJ483000* |
|  |  | *Triactis* | *producta* | KUNHM | EU490525 | ----------- | EU190876 | *KJ483125* | GU473350 |
|  | Amphianthidae | *Amphianthus* | sp. | USNM | FJ489413 | FJ489432 | FJ489450 | FJ489467 | FJ489502 |
|  |  | *Peronanthus* | sp**.** | AMNH | *KJ482917* | *KJ482956* | *KJ483014* | *KJ483066* | *KJ482976* |
|  | Andvakiidae | *Andvakia* | *boninensis* | KUNHM | EU190717 | EU190759 | EU190848 | *KJ483053* | FJ489479 |
|  |  | *Andvakia* | *discipulorum* | KUNHM | GU473273 | GU473287 | GU473316 | *KJ483051* | ------------- |
|  |  | *Telmatactis* | sp. | AMNH | JF832968 | JF832979 | *KJ483013* | *KJ483135* | ----------- |
|  | Antipodactinidae | *Antipodactis* | *awii* | AMNH | GU473271 | GU473286 | GU473303 | *KJ483074* | GU473337 |
|  | Bathyphelliidae | *Bathyphellia* | *australis* | KUNHM | FJ489402 | FJ489422 | EF589063 | EF589086 | FJ489482 |
|  | **Boloceroididae** | *Boloceroides* | *mcmurrichi* | KUNHM | GU473270 | ---------- | EU190852 | *KJ483103* | *KJ483002* |
|  |  | *Bunodeopsis* | *globulifera* | AMNH | *KJ482940* | *KJ482949* | *KJ483025* | *KJ483122* | *KJ482992* |
|  | Diadumenidae | *Diadumene* | *cincta* | KUNHM | EU190725 | EU190769 | EU190856 | *KJ483106* | FJ489490 |
|  |  | *Diadumene* | *leucolena* | KUNHM | JF832957 | JF832977 | JF832986 | *KJ483123* | JF833006 |
|  |  | *Diadumene* | sp. | KUNHM | JF832960 | JF832976 | JF832980 | *KJ483130* | JF833005 |
|  | **Galantheanthemidae** | *Galatheanthemum* | sp. nov. | NA | *KJ482918* | *KJ482955* | *KJ483012* | *KJ483065* | *KJ482977* |
|  |  | *Galatheanthemum* | *profundus* | AMNH | *KJ482919* | *KJ482954* | *KJ483011* | *KJ483119* | *KJ482978* |
|  | **Gonatiniidae** | *Gonactinia* | *prolifera* (Chile) | AMNH | *KJ482935* | *-------------* | *KJ483008* | *KJ483112* | *KJ482994* |
|  |  | *Gonactinia* | *prolifera* (USA) | AMNH | *KJ482937* | *KJ482969* | *KJ483009* | *KJ483077* | *KJ482995* |
|  |  | *Protantea* | *simplex* | AMNH | *KJ482939* | *KJ482970* | *KJ483010* | *KJ483078* | *KJ482993* |
|  | Halcampidae | *Cactosoma* | sp. nov. | AMNH | GU473279 | GU473297 | GU473313 | GU473329 | GU473346 |
|  |  | *Halcampa* | *duodecimcirrata* | KUNHM | JF832966 | EU190776 | AF254375 | EU190820 | ------------- |
|  |  | *Halcampoides* | *purpurea* | AMNH | EU190735 | EU190780 | AF254380 | *KJ483100* | ------------- |
|  | Haliplanellidae | *Haliplanella* | *lineata* (USA) | KUNHM | EU190730 | EU190774 | EU190860 | *KJ483108* | FJ489506 |
|  |  | *Haliplanella* | *lineata* (Japan) | KUNHM | JF832965 | JF832973 | JF832987 | *KJ483107* | JF833007 |
|  | Hormathiidae | *Actinauge* | *richardi* | KUNHM | EU190719 | EU190761 | EU190850 | *KJ483055* | FJ489480 |
|  |  | *Adamsia* | *palliata* | KUNHM | FJ489398 | FJ489419 | FJ489436 | *KJ483101* | FJ489474 |
|  |  | *Allantactis* | *parasitica* | KUNHM | FJ489399 | FJ489420 | FJ489439 | *KJ483056* | FJ489478 |
|  |  | *Calliactis* | *japonica* | KUNHM | FJ489403 | FJ489423 | FJ489441 | *KJ483057* | FJ489486 |
|  |  | *Calliactis* | *parasitica* | KUNHM | EU190711 | EU190752 | EU190842 | *KJ483102* | FJ489475 |
|  |  | *Calliactis* | *polypus* | KUNHM | FJ489407 | FJ489427 | FJ489445 | *KJ483058* | FJ489485 |
|  |  | *Calliactis* | *tricolor* | KUNHM | FJ489405 | FJ489425 | FJ489443 | *KJ483059* | FJ489488 |
|  |  | *Chondrophellia* | orangina | USNM | FJ489406 | FJ489426 | FJ489444 | *KJ483060* | FJ489489 |
|  |  | *Hormathia* | *armata* | AMNH | EU190731 | EU190775 | EU190861 | *KJ483062* | FJ489491 |
|  |  | *Hormathia* | *lacunifera* | AMNH | FJ489409 | FJ489428 | FJ489446 | *KJ483063* | FJ489492 |
|  |  | *Hormathia* | *pectinata* | AMNH | FJ489415 | FJ489430 | FJ489448 | FJ489465 | FJ489497 |
|  |  | *Paracalliactis* | *japonica* | CMHN | FJ489411 | FJ489429 | FJ489447 | *KJ483061* | FJ489496 |
|  |  | *Paraphelliactis* | sp. | KUNHM | FJ489412 | FJ489431 | FJ489449 | FJ489466 | FJ489498 |
|  | Isanthidae | *Isanthus* | *capensis* | AMNH | JF832967 | GU473291 | GU473307 | *KJ483096* | GU473362 |
|  |  | *Isoparactis* | *fabiani* | AMNH | JF832964 | GU473283 | GU473300 | *KJ483124* | GU473355 |
|  | Kadosactinidae | *Alvinactis* | *chessi* | USNM | GU473278 | GU473296 | GU473312 | *KJ483052* | GU473352 |
|  |  | *Cyananthea* | *hourdezi* | USMN | GU473275 | GU473293 | GU473309 | *KJ483081* | GU473364 |
|  |  | *Jasonactis* | *erythraios* | USNM | ------------- | GU473289 | GU473305 | *KJ483079* | GU473339 |
|  |  | *Kadosactis* | *antarctica* | AMNH | FJ489410 | EU190782 | EU190865 | *KJ483080* | FJ489504 |
|  | Metridiidae | *Metridium* | *s. lobatum* | KUNHM | JF832962 | JF832971 | JF832981 | *KJ483114* | JF833002 |
|  |  | *Metridium* | *senile* (WA) | KUNHM | EU190740 | EU190786 | AF052889 | *KJ483076* | FJ489494 |
|  |  | *Metridium* | *senile* (ME) | AMNH | *KJ482916* | *KJ482950* | *KJ483035* | *KJ483113* | *KJ482975* |
|  | Nemathidae | *Nemanthus* | *nitidus* | KUNHM | EU190741 | EU190787 | EU190868 | *KJ483064* | FJ489495 |
|  | Ostiactinidae | *Ostiactis* | *pearseae* | CAS | EU190751 | EU190798 | EU190878 | *KJ483082* | GU473365 |
|  | Phelliidae | *Phellia* | *gausapata* | ZSM | EU190744 | EU190790 | EU190870 | *KJ483054* | FJ489473 |
|  |  | *Phellia* | *exlex* | KUNHM | JF832958 | JF832978 | JF832984 | *KJ483121* | JF833004 |
|  | Sagartiidae | *Actinothoe* | *sphyrodeta* | ZSM | FJ489401 | FJ489421 | FJ489440 | *KJ483111* | FJ489481 |
|  |  | *Anthothoe* | *chilensis* | ZSM | FJ489397 | FJ489416 | FJ489434 | FJ489453 | FJ489470 |
|  |  | *Cereus* | *pedunculatus* | KUNHM | EU190724 | EU190767 | EU190855 | EU190813 | FJ489471 |
|  |  | *Cereus* | *herpetodes* | KUNHM | JF832956 | JF832969 | JF832983 | JF832992 | ----------- |
|  |  | *Sagartia* | *elegans* | KUNHM | ------------- | ---------- | JF832989 | JF832994 | JF833012 |
|  |  | *Sagartia* | *troglodytes* | KUNHM | EU190746 | EU190792 | EU190872 | *KJ483073* | FJ489499 |
|  |  | *Sagartia* | *ornata* | AMNH | JF832959 | JF832975 | JF832985 | *KJ483069* | JF833011 |
|  |  | *Sagartiogeton* | *laceratus* | KUNHM | EU190748 | EU190794 | EU190874 | *KJ483071* | FJ489500 |
|  |  | *Sagartiogeton* | *undatus* | KUNHM | FJ489400 | FJ489417 | FJ489435 | *KJ483070* | FJ489472 |
|  |  | *Verrillactis* | *paguri* | KUNHM | FJ489414 | FJ489433 | FJ489440 | *KJ483046* | FJ489503 |
| Antipatharia |  |  |  |  |  |  |  |  |  |
|  | Aphanipathidae | *Acanthopathes* | *thyoides* | USMNH | ----------- | FJ376986 | FJ389896 | FJ626238 | FJ381654 |
|  |  | *Elatopathes* | *abietina** | USMNH | ----------- | FJ376989 | FJ389894 | FJ626233 | KF054437 |
|  |  | *Phanopathes* | *expansa* | USMNH | ----------- | FJ376987 | FJ389897 | FJ626242 | FJ381655 |
|  |  | *Aphanipathes* | *verticillata mauiensis* | NA | ----------- | ----------- | KF054359 | KF054361 | KF054458 |
|  | Antipathidae | *Antipathes* | *griggi** | NA | ----------- | FJ376997 | FJ389904 | FJ429304 | GU296504 |
|  |  | *Antipathes* | *atlantica* | USMNH | ----------- | FJ376985 | FJ389895 | FJ626239 | HM060616 |
|  |  | *Cirrhipathes* | *anguina** | NA | ----------- | *KJ482973* | FJ389905 | FJ626243 | HM060614 |
|  |  | *Stichopathes* | cf. *dissimilis** | NA | ----------- | FJ376996 | FJ626245 | FJ626234 | KF054420 |
|  |  | *Stichopathes* | cf. *flagellum* | NA | ----------- | FJ376995 | FJ389903 | FJ626232 | FJ381660 |
|  | Cladopathidae | *Chrysopathes* | *formosa* | NA | DQ304771 | DQ304771 | ----------- | ----------- | DQ304771 |
|  |  | *Trissopathes* | *pseudotristicha** | USMNH | ----------- | FJ376991 | FJ389899 | FJ429305 | KF054409 |
|  | Leiopathidae | *Lei­­opathes* | *glaberrima** | NA | FJ597644 | FJ597644 | FJ389898 | FJ626241 | FJ597644 |
|  | Myriopathidae | *Tanacetipathes* | *barbadensis* | USMNH | ----------- | FJ376988 | FJ626244 | FJ626240 | FJ381650 |
|  | Schizopathidae | *Dendrobathypathes* | *boutillieri* | NA | ----------- | FJ376992 | FJ389900 | FJ626236 | FJ381651 |
|  |  | *Parantipathes* | cf. *hirondelle** | NA | ----------- | FJ376994 | FJ389902 | FJ626235 | ----------- |
|  |  | *Stauropathes* | cf. *punctata** | NA | ----------- | FJ376993 | FJ389901 | FJ626237 | FJ381657 |
| Ceriantharia |  |  |  |  |  |  |  |  |  |
|  | Arachnactinidae | *Isarachnanthus* | *nocturnus* | NA | ----------- | JX125669 | AB859826 | AB859832 | *KJ482980* |
|  | Cerianthidae | *Ceriantheomorphe* | *brasiliensis* | NA | *KJ482914* | JF915193 | AB859823 | AB859831 | *KJ482974* |
|  |  | *Pachycerianthus* | sp. | NA | *KJ482915* | ----------- | AB859829 | AB859833 | ----------- |
| Corallimorpharia |  |  |  |  |  |  |  |  |  |
|  | Corallimorphidae | *Corallimorphus* | *profundus* | AMNH | *KJ482941* | *KJ482972* | *KJ483027* | *KJ483129* | ----------- |
|  | Corynactinidae | *Corynactis* | *viridis* | NA | EF597099 | EF589058 | EF589065 | *KJ483041* | ----------- |
|  | Ricordeidae | *Ricordea* | *florida* | NA | *KJ482913* | EF589057 | EF589067 | *KJ483045* | DQ640648 |
| Octocorallia |  |  |  |  |  |  |  |  |  |
|  | Briareidae | *Briareum* | *asbestinum* | RMNH | DQ640649 | DQ640649 | KF992837 | KF992839 | DQ640649 |
|  | Gorgoniidae | *Antillogorgia* | *bipinnata* | RMNH | DQ640646 | NC008157 | KJ411642 | KJ411643 | DQ640646 |
|  | Nephtheidae | *Dendronephthya* | *sinaiensis** | RMNH | FJ372991 | FJ372991 | KF992836 | KF992838 | FJ372991 |
| Scleractinia |  |  |  |  |  |  |  |  |  |
|  | Agariciidae | *Pavona* | *varians** | NA | EF597083 | *KJ482943* | AF052883 | EU262847 | NC008165.1 |
|  | Caryophylliidae | *Phyllangia* | *mouchezii* | GB | EF597022 | AF265605 | AF052887 | EU262798 | ----------- |
|  |  | *Thalamophyllia* | *riisei** | GB | EF597087 | AF265590 | ----------- | EU262868 | ----------- |
|  | Dendrophylliidae | *Tubastraea* | *coccinea* | NA | EF597045 | *KJ482948* | AJ133556 | EU262864 | ----------- |
|  | Faviidae | *Montastraea* | *franksi* | NA | EF597010 | *KJ482947* | AY026382 | AY026375 | NC007225.1 |
|  | Fungiacyathidae | *Fungiacyathus* | *marenzelleri* | NA | EF597074 | *XXXXXXX* | EF589074 | EU262862 | ----------- |
|  | Pocilloporidae | *Madracis* | *mirabilis* | GB | NC011160 | NC011160 | AY950684 | EU262845 | ----------- |
|  |  | *Meandrina* | *meandrites* | NA | EF597032 | ----------- | *KJ483005* | EU262815 | ----------- |
|  |  | *Pocillopora* | *meandrina** | NA | EF596977 | *KJ482945* | *KJ483006* | EU262803 | NC009798.1 |
|  | Siderastreidae | *Siderastrea* | *siderea** | NA | EF597067 | *KJ482944* | *KJ483007* | EU262848 | NC008167 |
| Zoanthidea |  |  |  |  |  |  |  |  |  |
|  | Epizoanthidae | *Epizoanthus* | *illoricatus* | NA | AY995901 | EU591597 | KC218424 | *KJ483036* | ----------- |
|  |  |  | *paguricola* | NA | AY995902 | AY995928 | KC218427 | *KJ483042* | ----------- |
|  |  |  | *scotinus* | NA | GQ464967 | ----------- | KC218425 | *KJ483043* | ----------- |
|  |  | *Hydrozoanthus* | *gracilis* | NA | GQ464953 | AY995942 | *KJ483003* | *KJ483038* | ----------- |
|  |  |  | *tunicans* | MNHG | GQ464955 | EU828760 | *KJ483004* | *KJ483039* | ----------- |
|  | Parazoanthidae | *Parazoanthus* | *axinellae* | NA | GQ464940 | EU828754 | KC218416 | *KJ483044* | ----------- |
|  |  |  | *puertoricense* | NA | AY995916 | EU828758 | KC218418 | *KJ483037* | ----------- |
|  |  |  | *swiftii* | NA | GQ464945 | EU828755 | KC218417 | *KJ483040* | ----------- |
|  |  | *Savalia* | *savaglia* | GB | AY995905 | DQ825686 | HM044299 | HM044298 | DQ825686 |
| Hexacorallia *incertis ordinis* | **Relicanthidae** | *Relicanthus* | *daphneae* | FMNH | *KJ482934* | *KJ482971* | *KJ483028* | *KJ483131* | *KJ482999* |

Taxa are organized alphabetically within their family after this study. Bold type indicates taxa whose position/composition has been amended, see text and Appendix 1. New sequences indicate in italics. (*) sequences from specimens of different species. AMNH: American Museum of Natural History; CAS: California Academy of Sciences; FMNH: Field Museum of Natural History; KUNHM: University of Kansas Natural History Museum; MNHG: Museum of Natural History of Geneva; RMNH: Rijksmuseum van Natuurlijke Historie; USNM: U. S. National Museum of Natural History; ZSM: Bavarian State Collection of Zoology; GB: sequence from Genbank, refer to Genbank for voucher information; NA: voucher not available.
