# Supplementary material for: Hidden among Sea Anemones: The First Comprehensive Phylogenetic Reconstruction of the Order Actiniaria (Cnidaria, Anthozoa, Hexacorallia) Reveals a Novel Group of Hexacorals
Source: PLoS One. 2014 May 7;9(5):e96998. doi: 10.1371/journal.pone.0096998 (PMC4013120; doi:10.1371/journal.pone.0096998)
Supplement: Table S2 — Parameters implemented within MrBayes v.3.1.2. (DOCX) [file pone.0096998.s002.docx]

**Table S2. Parameters implemented within MrBayes v.3.1.2.**

| **Gene Region** | **# Taxa / Characters** | **Gamma / Pinvar** | **Generations (million)** | **# Generations discarded** | **Average SD Split Frequencies** |
| --- | --- | --- | --- | --- | --- |
| Mt 12S | 135 / 830 | 0.5280 | 10 | 2,500 | 0.012567 |
| Mt 16S | 146 / 472 | 0.4900 | 10 | 2,500 | 0.014116 |
| Mt *cox3* | 127 / 722 | 0.3140 | 10 | 2,500 | 0.011298 |
| Nc 18S | 151 / 1684 | 0.3630 / 0.4140 | 40 | 10,000 | 0.018103 |
| Nc 28S | 150 / 3067 | 0.4150 / 0.4140 | 40 | 10,000 | 0.020446 |
| *NcContig* | 153 / 4751 | Partitioned | 80 | 2,000 | 0.049843 |

All analyses utilized 2 runs, 4 chains and had a sample frequency of 1,000 (exception: *NcContig*: 10,000). Model of nucleotide substitution: GTR + G (12S, 16S, cox3); GTR+I+G (18S, 28S). Mt: mitochondrial. Nc: nuclear. #: number. Pinvar: proportion of invariable sites. SD: standard deviation. The *NcContig* dataset was partitioned.
