# Supplementary material for: Hidden among Sea Anemones: The First Comprehensive Phylogenetic Reconstruction of the Order Actiniaria (Cnidaria, Anthozoa, Hexacorallia) Reveals a Novel Group of Hexacorals
Source: PLoS One. 2014 May 7;9(5):e96998. doi: 10.1371/journal.pone.0096998 (PMC4013120; doi:10.1371/journal.pone.0096998)
Supplement: Table S3 — Parameters implemented in MrBayes v.3.1.2. (DOCX) [file pone.0096998.s003.docx]

**Table S3. Parameters implemented in MrBayes v.3.1.2.**

| **Gene Region** | **# Taxa / Characters** | **Gens (million)** | **Sample Frequency** | **# Runs / Chains** | **# Swaps / Heating** | **# Generations discarded** | **Ave SD Split Frequencies** |
| --- | --- | --- | --- | --- | --- | --- | --- |
| *MtContig* | 156 / 2024 | 50 | 1,000 | 2 / 4 | 1 / 0.1 | 12,500 | 0.115447 |
| *MtContig* | 156 / 2024 | 50 | 10,000 | 2 / 6 | 1 / 0.1 | 1,250 | 0.105748 |
| *MtContig* | 156 / 2024 | 50 | 10,000 | 2 / 6 | 3 / 0.2 | 1,250 | 0.124020 |
| *MasterContig* | 156 / 6775 | 30 | 1,000 | 2 / 4 | 1 / 0.1 | 7,500 | 0.135119 |
| *MasterContig* | 156 / 6775 | 60 | 10,000 | 2 / 4 | 1 / 0.1 | 1,500 | 0.141684 |

Datasets for which chains did not converge using MrBayes v.3.1.2. Mt: mitochondrial. Nc: nuclear. Gens: generations. Ave: average. SD: standard deviation. #: number. The *MtContig* and *MasterContig* datasets were partitioned.
