# Supplementary material for: Hidden among Sea Anemones: The First Comprehensive Phylogenetic Reconstruction of the Order Actiniaria (Cnidaria, Anthozoa, Hexacorallia) Reveals a Novel Group of Hexacorals
Source: PLoS One. 2014 May 7;9(5):e96998. doi: 10.1371/journal.pone.0096998 (PMC4013120; doi:10.1371/journal.pone.0096998)
Supplement: Table S4 — Parameters implemented in PhyloBayes v3.3b. (DOCX) [file pone.0096998.s004.docx]

**Table S4. Parameters implemented in PhyloBayes v3.3b.**

| **Gene Region** | **# Taxa / Characters** | **# Cycles** | **Burn-in** | **Maxdiff** | **Meandiff** |
| --- | --- | --- | --- | --- | --- |
| Mt 12S | 135 / 830 | 35,352-35,446 | 10% (3,535) | 0.0670527 | 0.00260182 |
| Mt 16S | 146 / 472 | 20,623-20,634 | 10% (2,062) | 0.075431 | 0.00341621 |
| Mt *cox*3 | 127 / 722 | 144,049-144,915 | 10% (14,492) | 0.0818219 | 0.00238712 |
| Nc 28S | 150 / 3067 | 75,495-75,639 | 5% (3,782) | 0.292268 | 0.00526072 |

Model: CAT + GTR. 4 chains. After burn-in, we sampled every 10 trees thereafter. Mt: mitochondrial. Nc: nuclear. Maxdiff: largest discrepancy observed across all bipartitions. Meandiff: mean discrepancy observed across all bipartitions. #: number.
