# Supplementary material for: Hidden among Sea Anemones: The First Comprehensive Phylogenetic Reconstruction of the Order Actiniaria (Cnidaria, Anthozoa, Hexacorallia) Reveals a Novel Group of Hexacorals
Source: PLoS One. 2014 May 7;9(5):e96998. doi: 10.1371/journal.pone.0096998 (PMC4013120; doi:10.1371/journal.pone.0096998)
Supplement: Table S5 — Ancestral state reconstruction of seven morphological characters. (DOCX) [file pone.0096998.s005.docx]

**Table S5. Ancestral state reconstruction of seven morphological characters.**

| **Genus** | **Species** | **Apical flaps** | **Basilar muscles** | **Marginal sphincter muscle** | **Acontia** | **Deciduous tentacles** | **Endosymbiosis** | **Longitudinal ectodermal muscles in column** |
| --- | --- | --- | --- | --- | --- | --- | --- | --- |
| *Acanthopathes* | *thyoides* | 0 | 0 | 0 | 0 | 0 | 0 | 0 |
| *Actinauge* | *richardi* | 1 | 1 | 1 | 1 | 0 | 0 | 0 |
| *Actinernus* | *antarcticus* | 1 | 0 | 0 | 0 | 0 | 0 | 0 |
| *Actinia* | *fragacea* | 1 | 1 | 2 | 0 | 0 | 0 | 0 |
| *Actinoscyphia* | *plebeia* | 1 | 1 | 1 | 0 | 0 | 0 | 0 |
| *Actinostephanus* | *haeckeli* | 1 | 1 | 0 | 0 | 0 | 1 | 0 |
| *Actinostola* | *chilensis* | 1 | 1 | 1 | 0 | 0 | 0 | 0 |
| *Actinostola* | *crassicornis* | 1 | 1 | 1 | 0 | 0 | 0 | 0 |
| *Actinostola* | *georgiana* | 1 | 1 | 1 | 0 | 0 | 0 | 0 |
| *Actinothoe* | *sphyrodeta* | 1 | 1 | 1 | 1 | 0 | 1 | 0 |
| *Adamsia* | *palliata* | 1 | 1 | 1 | 1 | 0 | 0 | 0 |
| *Aiptasia* | *mutabilis* | 1 | 1 | 1 | 1 | 0 | 1 | 1 |
| *Aiptasia* | *pallida* | 1 | 1 | 1 | 1 | 0 | 1 | 1 |
| *Alicia* | *sansibarensis* | 1 | 1 | 0 | 0 | 0 | 1 | 1 |
| *Allantactis* | *parasitica* | 1 | 1 | 1 | 1 | 0 | 0 | 0 |
| *Alvinactis* | *chessi* | 1 | 1 | 1 | 0 | 0 | 0 | 0 |
| *Andvakia* | *boninensis* | 1 | 0 | 1 | 1 | 0 | 0 | 0 |
| *Andvakia* | *discipulorum* | 1 | 0 | 1 | 1 | 0 | 0 | 0 |
| *Anemonia* | *viridis* | 1 | 1 | 2 | 0 | 0 | 1 | 0 |
| *Antholoba* | *achates* | 1 | 1 | 1 | 0 | 0 | 0 | 0 |
| *Anthopleura* | *elegantissima* | 1 | 1 | 2 | 0 | 0 | 1 | 0 |
| *Anthosactis* | *janmayeni* | 1 | 1 | 1 | 0 | 0 | 0 | 0 |
| *Anthostella* | *stephensoni* | 1 | 1 | 2 | 0 | 0 | 0 | 0 |
| *Anthothoe* | *chilensis* | 1 | 1 | 1 | 1 | 0 | 0 | 0 |
| *Antillogorgia* | *bipinnata* | 0 | 0 | 0 | 0 | 0 | 1 | 0 |
| *Aphanipathes* | *verticillata mauiensis* | 0 | 0 | 0 | 0 | 0 | ? | 0 |
| *Antipathes* | *atlantica* | 0 | 0 | 0 | 0 | 0 | 1 | 0 |
| *Antipathes* | *griggi* | 0 | 0 | 0 | 0 | 0 | 1 | 0 |
| *Antipodactis* | *awii* | 1 | 1 | 1 | 1 | 0 | 0 | 0 |
| *Amphianthus* | sp. | 1 | 1 | 1 | 1 | 0 | 0 | 0 |
| *Bartholomea* | *annulata* | 1 | 1 | 1 | 1 | 0 | 1 | 1 |
| *Bathyphellia* | *australis* | 1 | 1 | 1 | 1 | 0 | 0 | 0 |
| *Bolocera* | *kerguelensis* | 1 | 1 | 2 | 0 | 1 | 0 | 0 |
| *Boloceroides* | *mcmurrichi* | 1 | 0 | 0 | 0 | 1 | 1 | 1 |
| *Briareum* | *asbestinum* | 0 | 0 | 0 | 0 | 0 | 1 | 0 |
| *Bunodactis* | *verrucosa* | 1 | 1 | 2 | 0 | 0 | 0 | 0 |
| *Bunodeopsis* | *globulifera* | 1 | 0 | 0 | 0 | 1 | 1 | 1 |
| *Bunodosoma* | *grandis* | 1 | 1 | 2 | 0 | 0 | 0 | 0 |
| *Cactosoma* | sp. nov. | 1 | 0 | 1 | 0 | 0 | 0 | 0 |
| *Calliactis* | *japonica* | 1 | 1 | 1 | 1 | 0 | 0 | 0 |
| *Calliactis* | *parasitica* | 1 | 1 | 1 | 1 | 0 | 0 | 0 |
| *Calliactis* | *polypus* | 1 | 1 | 1 | 1 | 0 | 1 | 0 |
| *Calliactis* | *tricolor* | 1 | 1 | 1 | 1 | 0 | 0 | 0 |
| *Capnea* | *georgiana* | 1 | 1 | 2 | 0 | 0 | 0 | 0 |
| *Cereus* | *herpetodes* | 1 | 1 | 1 | 1 | 0 | 0 | 0 |
| *Cereus* | *pedunculatus* | 1 | 1 | 1 | 1 | 0 | 1 | 0 |
| *Ceriantheomorphe* | *brasiliensis* | 0 | 0 | 0 | 0 | 0 | 0 | 1 |
| *Chondrophellia* | *orangina* | 0 | 1 | 1 | 1 | 0 | 0 | 0 |
| *Chrysopathes* | *formosa_* | 0 | 0 | 0 | 0 | 0 | 0 | 0 |
| *Cirrhipathes* | *anguina* | 0 | 0 | 0 | 0 | 0 | 1 | 0 |
| *Corallimorphus* | *profundus* | 0 | 0 | 0 | 0 | 0 | 0 | 0 |
| *Corynactis* | *viridis* | 0 | 0 | 0 | 0 | 0 | 1 | 0 |
| *Cyananthea* | *hourdezi* | 1 | 1 | 1 | 0 | 0 | 0 | 0 |
| *Dactylanthus* | *antarcticus* | 1 | 0 | 0 | 0 | 0 | 0 | 0 |
| *Dendrobathypathes* | *boutillieri* | 0 | 0 | 0 | 0 | 0 | 0 | 0 |
| *Dendronephthya* | *sinaiensis* | 0 | 0 | 0 | 0 | 0 | 0 | 0 |
| *Diadumene* | *cincta* | 1 | 1 | 0 | 1 | 0 | 0 | 0 |
| *Diadumene* | *leucolena* | 1 | 1 | 0 | 1 | 0 | 0 | 0 |
| *Diadumene* | sp. | 1 | 1 | 0 | 1 | 0 | 0 | 0 |
| *Edwardsia* | *elegans* | 1 | 0 | 0 | 0 | 0 | 0 | 0 |
| *Edwardsia* | *japonica* | 1 | 0 | 0 | 0 | 0 | 0 | 0 |
| *Edwardsia* | *timida* | 1 | 0 | 0 | 0 | 0 | 0 | 0 |
| *Edwardsianthus* | *gilbertensis* | 1 | 0 | 0 | 0 | 0 | 0 | 0 |
| *Elatopathes* | *abientina* | 0 | 0 | 0 | 0 | 0 | ? | 0 |
| *Epiactis* | *lisbethae* | 1 | 1 | 2 | 0 | 0 | 0 | 0 |
| *Epizoanthus* | *illoricatus* | 0 | 0 | 1 | 0 | 0 | ? | 0 |
| *Epizoanthus* | *paguricola* | 0 | 0 | 1 | 0 | 0 | ? | 0 |
| *Epizoanthus* | *scotinus* | 0 | 0 | 1 | 0 | 0 | ? | 0 |
| *Fungiacyathus* | *marenzelleri* | 0 | 0 | 0 | 0 | 0 | 0 | 0 |
| *Galatheanthemum* | sp. | 1 | 0 | 1 | 0 | 0 | 0 | 0 |
| *Galatheanthemum* | *profundus* | 1 | 0 | 1 | 0 | 0 | 0 | 0 |
| *Glyphoperidium* | *bursa* | 1 | 1 | 2 | 0 | 0 | 0 | 0 |
| *Gonactinia* | *prolifera* (Chile) | 1 | 0 | 0 | 0 | 0 | 0 | 1 |
| *Gonactinia* | *prolifera* (USA) | 1 | 0 | 0 | 0 | 0 | 0 | 1 |
| *Halcampa* | *duodecimcirrata* | 1 | 0 | 1 | 0 | 0 | 0 | 0 |
| *Halcampoides* | *purpurea* | 1 | 0 | 0 | 0 | 0 | 0 | 0 |
| *Halcurias* | *pilatus* | 1 | 0 | 1 | 0 | 0 | 0 | 0 |
| *Haliplanella* | *lineata* (Japan) | 1 | 1 | 0 | 1 | 0 | 0 | 0 |
| *Haliplanella* | *lineata* (USA) | 1 | 1 | 0 | 1 | 0 | 0 | 0 |
| *Haloclava* | *producta* | 1 | 0 | 0 | 0 | 0 | 0 | 0 |
| *Haloclava* | sp. | 1 | 0 | 0 | 0 | 0 | 0 | 0 |
| *Harenactis* | *argentina* | 1 | 0 | 0 | 0 | 0 | 0 | 0 |
| *Heteractis* | *magnifica* | 1 | 1 | 2 | 0 | 0 | 1 | 0 |
| *Hormathia* | *armata* | 1 | 1 | 1 | 1 | 0 | 0 | 0 |
| *Hormathia* | *lacunifera* | 1 | 1 | 1 | 1 | 0 | 0 | 0 |
| *Hormathia* | *pectinata* | 1 | 1 | 1 | 1 | 0 | 0 | 0 |
| *Hormosoma* | *scotti* | 1 | 1 | 1 | 0 | 0 | 0 | 0 |
| *Hydrozoanthus* | *gracilis* | 0 | 0 | 2 | 0 | 0 | 1 | 0 |
| *Hydrozoanthus* | *tunicans* | 0 | 0 | 2 | 0 | 0 | 1 | 0 |
| *Isactinernus* | *quadrolobatus* | 1 | 0 | 0 | 0 | 0 | 0 | 0 |
| *Isanthus* | *capensis* | 1 | 1 | 1 | 0 | 0 | 0 | 0 |
| *Isarachnanthus* | *nocturnus* | 0 | 0 | 0 | 0 | 0 | 0 | 1 |
| *Isoparactis* | *fabiani* | 1 | 1 | 1 | 1 | 0 | 0 | 0 |
| *Isosicyonis* | *alba* | 1 | 1 | 2 | 0 | 0 | 0 | 0 |
| *Isosicyonis* | *striata* | 1 | 1 | 2 | 0 | 0 | 0 | 0 |
| *Isotealia* | *antarctica* | 1 | 1 | 2 | 0 | 0 | 0 | 0 |
| *Jasonactis* | *erythraios* | 1 | 1 | 1 | 1 | 0 | 0 | 0 |
| *Kadosactis* | *antarctica* | 1 | 1 | 1 | 1 | 0 | 0 | 0 |
| *Korsaranthus* | *natalensis* | 1 | 1 | 2 | 0 | 0 | 0 | 0 |
| *Leiopathes* | *glaberrima _* | 0 | 0 | 0 | 0 | 0 | 0 | 0 |
| *Liponema* | *brevicornis* | 1 | 1 | 2 | 0 | 1 | 0 | 0 |
| *Liponema* | *multiporum* | 1 | 1 | 2 | 0 | 1 | 0 | 0 |
| *Macrodactyla* | *doreenensis* | 1 | 1 | 2 | 0 | 0 | 1 | 0 |
| *Madracis* | *mirabilis* | 0 | 0 | 0 | 0 | 0 | 1 | 0 |
| *Meandrina* | *meandrites* | 0 | 0 | 0 | 0 | 0 | 1 | 0 |
| *Metridium* | *senile* (WA) | 1 | 1 | 1 | 1 | 0 | 0 | 0 |
| *Metridium* | *senile* (ME) | 1 | 1 | 1 | 1 | 0 | 0 | 0 |
| *Metridium* | *s. lobatum* | 1 | 1 | 1 | 1 | 0 | 0 | 0 |
| *Montastraea* | *franksi* | 0 | 0 | 0 | 0 | 0 | 1 | 0 |
| *Nemanthus* | *nitidus* | 1 | 1 | 1 | 0 | 0 | ? | 0 |
| *Nematostella* | *vectensis* | 1 | 0 | 0 | 0 | 0 | 0 | 0 |
| *Neoaiptasia* | *morbilla* | 1 | 1 | 2 | 1 | 0 | 0 | 0 |
| *Ostiactis* | *pearseae* | 1 | 1 | 1 | 0 | 0 | 0 | 0 |
| *Pachycerianthus* | sp. | 0 | 0 | 0 | 0 | 0 | 0 | 1 |
| *Paracalliactis* | *japonica* | 1 | 1 | 1 | 1 | 0 | 0 | 0 |
| *Paraphelliactis* | sp. | 1 | 1 | 1 | 1 | 0 | 0 | 0 |
| *Paranthipathes* | sp. | 0 | 0 | 0 | 0 | 0 | 0 | 0 |
| *Paranthus* | *niveus* | 1 | 1 | 1 | 0 | 0 | 0 | 0 |
| *Parazoanthus* | *axinellae* | 0 | 0 | 2 | 0 | 0 | 0 | 0 |
| *Parazoanthus* | *puertoricense* | 0 | 0 | 2 | 0 | 0 | 0 | 0 |
| *Parazoanthus* | *swiftii* | 0 | 0 | 2 | 0 | 0 | 0 | 0 |
| *Pavona* | *varians* | 0 | 0 | 0 | 0 | 0 | 1 | 0 |
| *Peachia* | *cylindrica* | 1 | 0 | 0 | 0 | 0 | 0 | 0 |
| *Peronanthus* | sp. | 1 | 1 | 1 | 0 | 0 | 0 | 0 |
| *Phanopathes* | *expansa* | 0 | 0 | 0 | 0 | 0 | ? | 0 |
| *Phellia* | *exlex* | 1 | 1 | 1 | 1 | 0 | 0 | 0 |
| *Phellia* | *gausapata* | 1 | 1 | 1 | 1 | 0 | 0 | 0 |
| *Phyllangia* | *mouchezii* | 0 | 0 | 0 | 0 | 0 | 0 | 0 |
| *Phymanthus* | *loligo* | 1 | 1 | 0 | 0 | 0 | 1 | 0 |
| *Pocillopora* | *meandrina* | 0 | 0 | 0 | 0 | 0 | 1 | 0 |
| *Preactis* | *milliardae* | 1 | 0 | 0 | 0 | 0 | 0 | 0 |
| *Protantea* | *simplex* | 1 | 0 | 0 | 0 | 0 | 0 | 1 |
| *Relicanthus* | *daphneae* | 1 | 0 | 0 | 0 | 1 | 0 | 1 |
| *Ricordea* | *florida* | 0 | 0 | 0 | 0 | 0 | 1 | 0 |
| *Sagartia* | *elegans* | 1 | 1 | 1 | 1 | 0 | 0 | 0 |
| *Sagartia* | *ornata* | 1 | 1 | 1 | 1 | 0 | 1 | 0 |
| *Sagartia* | *troglodytes* | 1 | 1 | 1 | 1 | 0 | 0 | 0 |
| *Sagartiogeton* | *laceratus* | 1 | 1 | 1 | 1 | 0 | ? | 0 |
| *Sagartiogeton* | *undatus* | 1 | 1 | 1 | 1 | 0 | 0 | 0 |
| *Savalia* | *savaglia* | 0 | 0 | 2 | 0 | 0 | 1 | 0 |
| *Siderastrea* | *siderea* | 0 | 0 | 0 | 0 | 0 | 1 | 0 |
| *Stauropathes* | *punctata* | 0 | 0 | 0 | 0 | 0 | 0 | 0 |
| *Stephanthus* | *antarcticus* | 1 | 0 | 0 | 0 | 0 | 0 | 0 |
| *Stichopathes* | *dissimilis* | 0 | 0 | 0 | 0 | 0 | 0 | 0 |
| *Stichopathes* | *flagellum* | 0 | 0 | 0 | 0 | 0 | 0 | 0 |
| *Stomphia* | *didemon* | 1 | 1 | 1 | 0 | 0 | 0 | 0 |
| *Stomphia* | *selaginella* | 1 | 1 | 1 | 0 | 0 | 0 | 0 |
| *Synhalcurias* | *laevis* | 1 | 0 | 0 | 0 | 0 | 0 | 0 |
| *Tanacetipathes* | *barbadensis* | 0 | 0 | 0 | 0 | 0 | 0 | 0 |
| *Thalamophyllia* | *riisei* | 0 | 0 | 0 | 0 | 0 | 0 | 0 |
| *Telmatactis* | sp. | 1 | 1 | 1 | 1 | 0 | 1 | 0 |
| *Triactis* | *producta* | 1 | 1 | 0 | 0 | 0 | 1 | 1 |
| *Trissopathes* | *pseudotristicha* | 0 | 0 | 0 | 0 | 0 | 0 | 0 |
| *Tubastraea* | *coccinea* | 0 | 0 | 0 | 0 | 0 | 1 | 0 |
| *Urticina* | *coriacea* | 1 | 1 | 2 | 0 | 0 | 0 | 0 |
| *Verrillactis* | *paguri* | 1 | 1 | 1 | 1 | 0 | 0 | 0 |

(0): absent; (1) present; in all characters except in the marginal sphincter muscle, in which (1) indicates mesogleal and (2) indicates endodermal; (?): unknown.
