# Supplementary material for: Hidden among Sea Anemones: The First Comprehensive Phylogenetic Reconstruction of the Order Actiniaria (Cnidaria, Anthozoa, Hexacorallia) Reveals a Novel Group of Hexacorals
Source: PLoS One. 2014 May 7;9(5):e96998. doi: 10.1371/journal.pone.0096998 (PMC4013120; doi:10.1371/journal.pone.0096998)
Supplement: Table S6 — Tree statistics for the sensitivity analysis for the different parameter set values of POY analyses. (DOCX) [file pone.0096998.s006.docx]

**Table S6.** **Tree statistics for the sensitivity analysis for the different parameter set values of POY analyses.**

| **GAP** | **Ts/Tv** | **MRI** | **ILD** | ***MasterContig*** | **Mt 12S** | **Mt 16S** | **Nc 18S** | **Nc 28S** | **Mt *cox*3** |
| --- | --- | --- | --- | --- | --- | --- | --- | --- | --- |

| 1 | 1 | **0.04879** | 0.095640090 | 28785 | 3640 | 3204 | 3515 | 11615 | 4058 |
| --- | --- | --- | --- | --- | --- | --- | --- | --- | --- |
| 1 | 2 | 0.06209 | 0.102221424 | 44521 | 5727 | 5136 | 5332 | 17648 | 6127 |
| 1 | 4 | 0.05807 | 0.112190602 | 71227 | 9432 | 8536 | 8399 | 27657 | 9212 |
| 2 | 1 | 0.07584 | 0.113267305 | 37257 | 4888 | 4426 | 4311 | 14265 | 5147 |
| 2 | 2 | 0.06458 | 0.123243923 | 60433 | 8094 | 7376 | 6829 | 22496 | 8190 |
| 2 | 4 | 0.06610 | 0.133567580 | 106156 | 14331 | 13194 | 11715 | 38510 | 14227 |
| 4 | 1 | 0.09813 | 0.141350292 | 52048 | 7030 | 6384 | 5653 | 18454 | 7170 |
| 4 | 2 | 0.07077 | 0.155872827 | 89233 | 12186 | 10961 | 9396 | 30595 | 12186 |
| 4 | 4 | 0.07184 | 0.166305819 | 162580 | 22304 | 19859 | 16820 | 54356 | 22203 |

Parameter set values: gap and transition/transversion ratio (Ts/Tv) costs; the cost for opening a gap was set to 0 for all of these analyses. The parameter set that minimizes incongruence among the partitions is highlighted in bold. Tree costs/lengths and MRI values for each analysis are provided. *MasterContig*: combination of all molecular partitions.
