# Supplementary material for: Hidden among Sea Anemones: The First Comprehensive Phylogenetic Reconstruction of the Order Actiniaria (Cnidaria, Anthozoa, Hexacorallia) Reveals a Novel Group of Hexacorals
Source: PLoS One. 2014 May 7;9(5):e96998. doi: 10.1371/journal.pone.0096998 (PMC4013120; doi:10.1371/journal.pone.0096998)
Supplement: Text S1 — Details of material and methods. (DOCX) [file pone.0096998.s007.docx]

**Text S1. Details of material and methods**

Secondary structure (SS) alignments were produced for 12S, 18S and 28S. SS models for nuclear rRNA genes were inferred according to Gillespie et al. [1] from a broad sampling of all cnidarian orders (995 species for 18S and 517 species for 28S). For the 18S sequences, the secondary structure template was obtained from the European ribosomal RNA Database (http://bioinformatics.psb.ugent.be/webtools/rRNA/index.html). The model of Schnare et al. [2] was used for the 28S alignment. The template alignment for the 12S sequences had more restricted taxon sampling, including 137 species of scleractinian corals, four species of corallimopharians and two species of sea anemones and is based on the putative model proposed by Pont-Kingdon et al. [3] for Metridium senile. Structural features within the third domain of 12S were further refined according to Hickson et al. [4]. Sequences obtained in this study were manually aligned to the reference alignments using GeneDoc v.2.6.002 [5]. The putative secondary structures of regions that could not be confidently aligned to the templates were estimated using RNAalifold [6] and further refined using MFold [7]. Equivocal base pairing in stem regions was detected using a custom PERL script. Alignment issues were manually corrected in GeneDoc and recursively re-evaluated with aid of the script until no further refinement was possible. Regions showing length heterogeneity with respect to the structural model were discarded from the alignment, except when one or more stems were completely absent from a group of sequences, while showing no structural heterogeneity across the remainder of the alignment. Because such indels result in blocks of gaps and base pairs with identical sizes, these regions were kept in the alignments and gaps were treated as missing data in subsequent phylogenetic analyses.

Heuristic searches were also performed in POY v.4.1.3. [8] using a timed search. This is a default search strategy that combines Wagner tree building with multiple rounds of TBR branch swapping, parsimony ratchet [9] and tree fusing [10]. The datasets were analyzed separately to assess the pattern of relationships from each and in a combined analysis of all the molecular data. All analyses were performed on a parallel cluster computer. The analysis of each single-gene dataset involved two 6-hour timed searches, run on four processors. The search strategy for the combined dataset consisted of two 96-hour timed searches, run on 10 processors, with the trees subjected to additional rounds of tree fusing and swapping [fuse (iterations: 250), swap (trees: 100)]. Between each timed search, all topologically unique trees were stored in memory [select (unique)], until the last round, when only topologically unique optimal trees were kept [select ()]. Selecting all unique trees following each iteration, regardless of optimality, helps to avoid local optima. The strict consensus reflects all unique and optimal trees in memory.

We explored the sensitivity (*sensu* [11]) of the topology to alignment parameters including gap opening, insertion/deletion (indel), and transition/transversion costs. Three indel cost ratios (1, 2, 4) and three transversion/transition (tv/ts) cost ratios (1, 2, 4) were used. This approach requires testing for character congruence among the various partitions with the parameter set that minimized the incongruence among the partitions being considered optimal. We used the Meta Retention Index (MRI) [12], an extension of Farris’ [13] Retention Index, that corresponds to the RI applied to the fragments of the sequence data as opposed to individual nucleotides. In direct optimization, the sequence fragment is the character as opposed to the individual nucleotides; therefore, character congruence among the various partitions was subsequently measured using the MRI [12].

**Supplemental references**

1. Gillespie JJ (2004) Characterizing regions of ambiguous alignment caused by the expansion and contraction of hairpin-stem loops in ribosomal RNA molecules. Mol Phyl Evol 33: 936-943.
2. Schnare MN, Damberger SH, Gray MW, Gutell RR (1996) Comprehensive comparison of structural characteristics in eukaryotic cytoplasmic large subunit (23S- like) ribosomal RNA. J Mol Biol 256: 701-719.
3. Pont-Kingdon GA, Beagley CT, Okimoto R, Wolstenholme DR (1994) Mitochondrial DNA of the sea anemone *Metridium senile* (Cnidaria): prokaryote-like genes for tRNA-f-Met and small-subunit ribosomal RNA, and standard genetic specificities for AGR and ATA codons. J Mol Evol 39: 387-399.
4. Hickson RE, Simon C, Cooper A, Spicer GS, Sullivan J, et al. (1996) Conserved sequence motifs, alignment, and secondary structure for the third domain of animal 12S rRNA. Mol Biol Evol 13: 150-169.
5. Nicholas KB, Nicholas HB, Deerfield DW (1997) GeneDoc: analysis and visualization of genetic variation. EMBNEW NEWS: 14.
6. Hofacker IL (2003) Vienna RNA secondary structure server. Nucleic Acids Res 31: 3429-3431.
7. Zuker M (2003) Mfold Web server for nucleic acid folding and hybridization prediction. Nucleic Acids Res 31: 3406-3415.
8. Varón A, Sy Vinh L, Wheeler W (2009) POY version 4: phylogenetic analysis using dynamic homologies. Cladistics 26: 72-85.
9. Nixon KC (1999) The Parsimony ratchet, a new method for rapid parsimony analysis. Cladistics 15: 407-414.
10. Goloboff PA (1999) Analyzing large data sets in reasonable times: solutions for composite optima. Cladistics 15: 415-428.
11. Wheeler WC (1995) Sequence alignment, parameter sensitivity, and phylogenetic analysis of molecular data. Syst Biol 44: 321-331.
12. Wheeler WC, Aagesen L, Arango CP, Faivovich J, Grant T, et al. (2006) Dynamic Homology and Phylogenetic Systematics: A Unified Approach Using POY. New York: American Museum of Natural History. 365 p.
13. Farris JS (1989) The retention index and rescaled consistency index. Cladistics 5: 417-419.
